# Solution-grown BiI/BiI_3_ van der Waals heterostructures for sensitive X-ray detection

**DOI:** 10.1038/s41467-023-37297-z

**Published:** 2023-03-23

**Authors:** Renzhong Zhuang, Songhua Cai, Zengxia Mei, Huili Liang, Ningjiu Zhao, Haoran Mu, Wenzhi Yu, Yan Jiang, Jian Yuan, Shuping Lau, Shiming Deng, Mingyue Han, Peng Jin, Cailin Wang, Guangyu Zhang, Shenghuang Lin

**Affiliations:** 1 Songshan Lake Materials Laboratory, 523808 Dongguan, Guangdong P. R. China; 2 Fujian Provincial Key Laboratory of Welding Quality Intelligent Evaluation, Longyan University, Longyan, Fujian P. R. China; 3 Department of Applied Physics, The Hong Kong Polytechnic University, Hunghom, Kowloon, Hong Kong P. R. China; 4 Institute of Physics, Chinese Academy of Science, 100190 Beijing, P. R. China; 5 HAMAMATSU Photonics (China) Co., LTD., 100020 Beijing, P. R. China; 6 State Key Laboratory of Modern Optical Instrumentation, College of Optical Science and Engineering, Zhejiang University, Hangzhou, Zhejiang China

**Keywords:** X-rays, Two-dimensional materials

## Abstract

X-ray detectors must be operated at minimal doses to reduce radiation health risks during X-ray security examination or medical inspection, therefore requiring high sensitivity and low detection limits. Although organolead trihalide perovskites have rapidly emerged as promising candidates for X-ray detection due to their low cost and remarkable performance, these materials threaten the safety of the human body and environment due to the presence of lead. Here we present the realization of highly sensitive X-ray detectors based on an environmentally friendly solution-grown thick BiI/BiI_3_/BiI (Bi_x_I_y_) van der Waals heterostructure. The devices exhibit anisotropic X-ray detection response with a sensitivity up to 4.3 × 10^4^ μC Gy^−1^ cm^−2^ and a detection limit as low as 34 nGy s^−1^. At the same time, our Bi_x_I_y_ detectors demonstrate high environmental and hard radiation stabilities. Our work motivates the search for new van der Waals heterostructure classes to realize high-performance X-ray detectors and other optoelectronic devices without employing toxic elements.

## Abstract (web summary)

Many of the most sensitive X-ray detectors are based on toxic elements such as lead, limiting their safe applications. Here, the authors report the realization of sensitive X-ray detectors based on solution-grown thick BiI/BiI3/BiI van der Waals heterostructures, showing a detection limit down to 34 nGy s^−1^ and high stability.

## Introduction

High-sensitive X-ray detection requiring a low-dose rate is of particular importance to reduce the risks of cancer caused by repeated exposure to ionizing radiation in the fields of physical examination such as medical diagnosis and security inspection^1,2^. Therefore, it promotes the exploration of X-ray detectors to improve the sensitivity and reduce the detection limit. High-sensitivity and low detection limit require the X-ray detectors to possess high resistivity, high attenuation coefficient, low electron–hole formation energy (*ε*_pair_), and excellent charge collection ability. Here, “high resistivity” results in the selection of materials with a large bandgap to reduce the temperature-induced carrier excitation. Whereas “low *ε*_pair_” needs the target materials with a small bandgap to generate more electron–hole pairs by a single X-ray photon. Therefore, a medium bandgap between 1.5 and 3.0 eV is considered appropriate to balance the *ε*_pair_ and resistivity^3^. Nowadays, excellent semiconductors such as metal halide perovskites and CZT in forms of single crystal, polycrystalline or thick film with medium bandgap have been developed for high-sensitive room temperature X-ray detection. However, they are still limited by toxicity, stability, or cost^4–9^.

Apart from the mentioned semiconductors, BiI_3_ is also an alternatively promising material with a medium bandgap. As reported, BiI_3_ is a 2D-layered semiconductor that belongs to *R*$\bar{3}$*−148* space group with a strongly anisotropic crystal structure consists of I–Bi-I tri-layers stacked by weak van der Waals interactions^10,11^. Owing to the appropriate bandgap (1.67 eV), high density (5.8 g cm^−3^), high atomic number (Z_Bi_ = 83, Z_I_ = 53), and high resistivity (10^8^−10^13^ Ω cm), BiI_3_ is attractive for hard radiation detection and has achieved sensitive X-ray detection recent years^12–25^.

This article reports a van der Waals heterostructure of Bi_x_I_y_ that served as a high-sensitive X-ray detector. By analyzing the samples’ cross-sectional images by aberration-corrected scanning transmission electron microscopy (ac-STEM), we found the obtained Bi_x_I_y_ is composed of thick BiI_3_ layers (main) alternately stacked with new thin Bi-rich layers (minor) with a chemical formula of BiI. Namely, Bi_x_I_y_ presents a heterostructure formed by the stackings of BiI/BiI_3_/BiI, which leads to a dual bandgap. Benefited from the heterostructure, Bi_x_I_y_ exhibits a higher X-ray sensitivity than BiI_3_ single crystal. Moreover, like many halide perovskite single crystals, macrosize Bi_x_I_y_ can be grown using a low-cost, handy low-temperature solution method. These advantages together with other merits such as good X-ray attenuation efficiency and charge collection ability, high resistivity, environmentally friendly, good environmental and hard radiation stability make Bi_x_I_y_ be attractive as a potential competitor for high-sensitive room temperature X-ray detection.

## Results

### Bi_x_I_y_ grown in solution

**Fig. 1:**
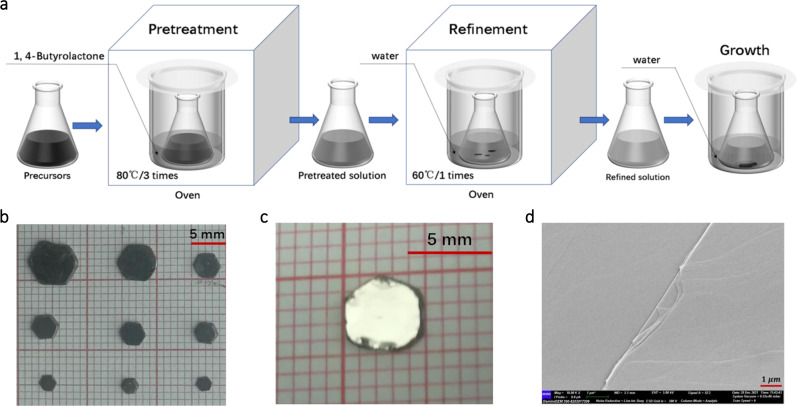
Preparation and characterization of Bi_x_I_y_. **a** A typical growth procedure for Bi_x_I_y_ includes solution pretreatment, solution refinement and crystal growth in room temperature water bath. **b** Optical image of as-grown crystals. **c** A typical as-grown crystal with the stripped surface. **d** SEM image of an exfoliated flake transferred onto a conductive tape.

The Bi_x_I_y_ were grown by a low-temperature solution technique, as shown in Fig. 1a. First, Bi_2_O_3_, I_2_, and Au were dissolved in a mixed hydroiodic acid and ethanol solution to form precursor solutions. Excessive iodine is used to dissolve gold in hydroiodic acid fully. Then the solution refinement technique was employed to grow high-quality crystals^26^. Namely, the precursor solutions were pretreated by solvothermal in 1,4-butyrolactone for three times and then refined by hydrothermal for 1-time. Bi^3+^ was reduced by I^−^ with Au acted as a catalyst during the solution pretreatment; the chemical reactions in the solution were:

1 $$4{{{{{{\rm{H}}}}}}}^{+}+4{{{{{{\rm{I}}}}}}}^{-}+{{{{{{\rm{O}}}}}}}_{2}\uparrow \mathop{\to }^{\triangle }2{{{{{{\rm{I}}}}}}}_{2}\uparrow+2{{{{{{\rm{H}}}}}}}_{2}{{{{{\rm{O}}}}}}$$

2 $${{{{{{\rm{I}}}}}}}_{2}+{{{{{{\rm{I}}}}}}}^{-} \mathop{\Leftrightarrow}^{ \triangle }{{{{{{\rm{I}}}}}}}_{3}^{-}$$

3 $${{{{{{\rm{Bi}}}}}}}^{3+}+2{{{{{{\rm{I}}}}}}}^{-} \mathop{\Leftrightarrow}^{ \triangle/{{{{{\rm{Au}}}}}}}{{{{{{\rm{Bi}}}}}}}^{+}+{{{{{{\rm{I}}}}}}}_{2}\uparrow$$

The iodine diffused into 1,4-butyrolactone and made it darken. Bi_x_I_y_ with regular hexagonal shape and size up to 6 × 6 × 1 mm^3^ were obtained from refined solution after 14 days of water bath growth at room temperature without any disturbance, as shown in Fig. 1b. More times pretreatment in 1,4-butyrolactone would promote massive nucleation. Smooth surfaces and Large flexible flakes of the grown Bi_x_I_y_ can be obtained by mechanical exfoliation, as shown in Fig. 1b and Supplementary Fig. 1. The morphology image (Fig. 1d) collected by scanning electron microscopy (SEM) reveals that the studied Bi_x_I_y_ flake possesses a high-quality exfoliated surface with no bubbles, holes, and inclusions. The morphology profile (Supplementary Fig. 1) of Bi_x_I_y_ flakes observed by atomic force microscopy (AFM) shows a layered structure. A step height of 0.66 nm was obtained and assigned to I–Bi–Bi–I monolayer (0.65 nm obtained by ac-STEM, see below), as shown in Supplementary Fig. 1.

Bi_x_I_y_ has the same X-ray diffraction (XRD) pattern (Supplementary Fig. 2) as BiI_3_, but exhibits broader peaks and preferred orientation along [001], indicates a softer nature of Bi_x_I_y_. The inductively coupled plasma atomic emission spectra (ICP-AES) result (35.2 wt.% Bi of as-grown Bi_x_I_y_) confirms the chemical composition is BiI_3_ (35.4 wt.% Bi in calculation). However, thermogravimetric (TG) and differential scanning calorimetry (DSC) analyses (Supplementary Fig. 3) show a different thermal behavior between Bi_x_I_y_ and BiI_3_. The melting point of Bi_x_I_y_ (408 °C) is slightly different from BiI_3_ (411 °C). BiI_3_ exhibits two decomposition temperatures above melting point at 417 °C and 431 °C, respectively. However, which are not observed in Bi_x_I_y_. As the temperature exceeding 411 °C, Bi_x_I_y_ remains 46.2% of its original mass, on the other hand BiI_3_ lost nearly all the mass. Meanwhile, no detectable mass loss is observed in the Bi_x_I_y_ even at a temperature of 300 °C, indicating its high thermal stability.

**Fig. 2:**
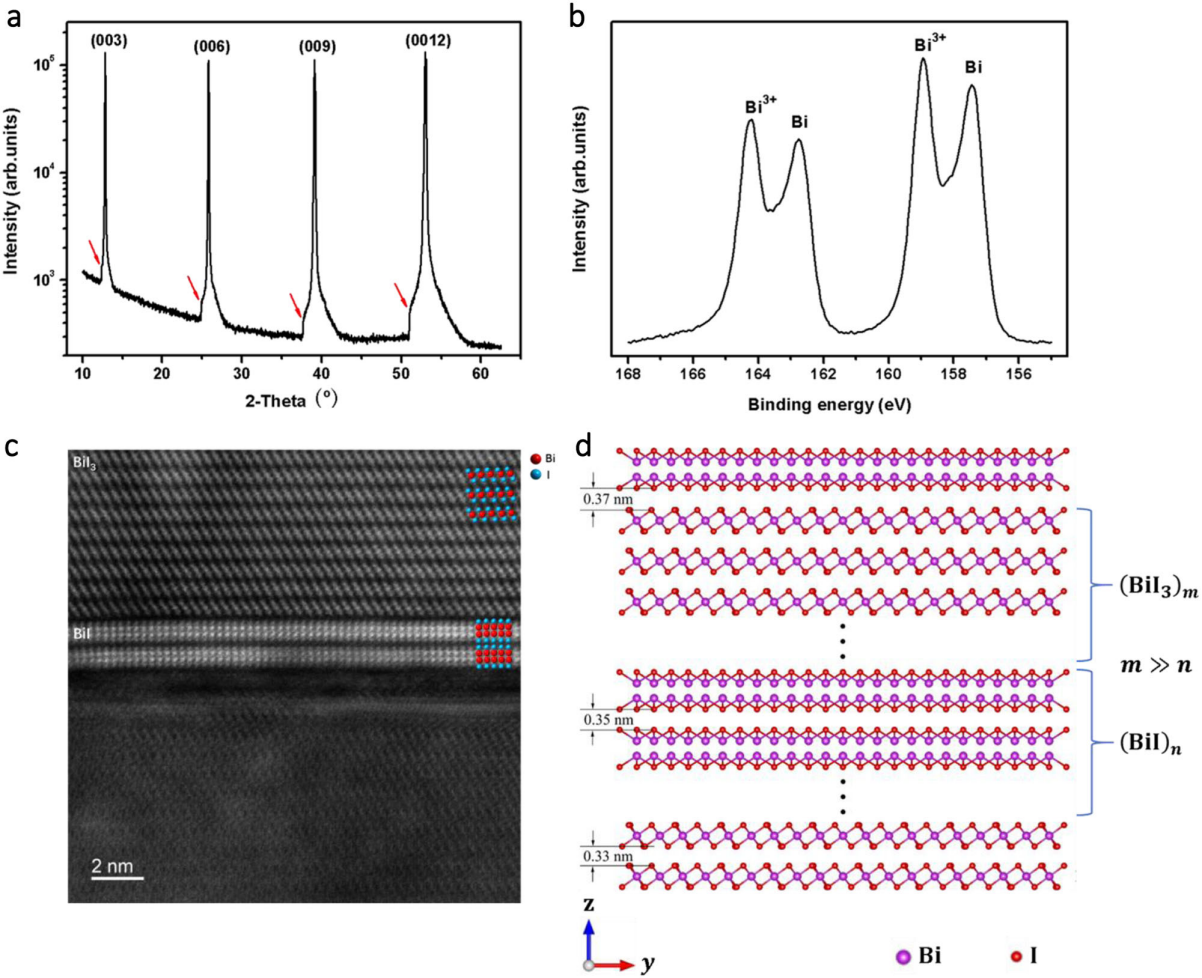
Structure characterization of Bi_x_I_y_. **a** (001) surface X-ray diffraction (XRD) pattern of Bi_x_I_y_, the red arrows point to a minor secondary diffraction of Bi_x_I_y_. **b** X-ray photoelectron spectroscopy (XPS) of Bi *4f* for Bi_x_I_y_. **c** High-angle annular dark-field scanning transmission electron microscope (HAADF-STEM) image of layered stacking of adjacent BiI_3_ and BiI in Bi_x_I_y_. **d** Side view of Bi_x_I_y_ along [100] direction, where *m* and *n* are integer values. The thickness of BiI and BiI_3_ layers is not controlled with atomic precision. The distance of BiI_3_/BiI, BiI/BiI, and BiI_3_/BiI_3_ are 0.37 nm, 0.35 nm and 0.33 nm, respectively.

The stripped surface examined by XRD shows a heterostructure character of Bi_x_I_y_ with a major diffraction of BiI_3_ (00k) and a regular minor secondary diffraction, as shown in Fig. 2a. The X-ray photoelectron spectroscopy (XPS) survey of Bi *4f* in the freshly stripped smooth surface of Bi_x_I_y_ shows the expected Bi^3+^ peaks^27^ at binding energies (BE) of 164.4 eV and 159.1 eV together with a distinct additional component shifted by 1.5 eV toward lower BE (Fig. 2b), assigned as Bi^+^ (see below). The dramatic change of valance band BE from BiI_3_ to Bi_x_I_y_ indicates a significant difference in the energy band between BiI_3_ and the Bi_x_I_y_, as shown in Supplementary Fig. 4.

Then we used aberration-corrected STEM to observe the stacking sequence of Bi_x_I_y_ by a high-angle annular dark-field (HAADF) imaging mode, which provides directly interpretable Z-contrast images at the atomic level^28,29^. Supplementary Fig. 5 shows several cross-sectional STEM images of flakes with different thicknesses exfoliated from a grown crystal. As seen in Supplementary Fig. 5, the flakes show alternate stacking of thin bright layers and thick dark layers. The bright layers are assigned to a Bi-rich phase due to the Z-contrast HAADF image^28,29^. It should be noted here that the thickness of the exfoliated Bi_x_I_y_ is mainly dependent on the middle part of BiI_3_. The STEM-EDS mapping results in Supplementary Fig. 5 also confirm a higher concentration of Bi in the bright layers. Detailed Z-contrast images of Bi-rich phase and the dark layers are shown in Supplementary Fig. 6. Bi-rich phase exhibits a layered van der Waals structure built by the stacking of I–Bi–Bi–I four atomic layers with a chemical composition of BiI. On the other hand, the dark layers have a BiI_3_ structure characterized by the staking of I–Bi–I three atomic layers^23^, which is consistent with the BiI_3_ atomic structure model. BiI_3_ and BiI layers are also held together by weak van der Waals force in the Bi_x_I_y_ structure, as shown in Fig. 2c. The STEM images clearly confirm the van der Waals heterostructure of Bi_x_I_y_ constructed by stacking of thick BiI_3_ and thin BiI layers, as shown in Fig. 2d.

The BiI layers could be separated from the Bi_x_I_y_ by mechanical exfoliation. As a result, we successfully obtained the thinnest BiI film composed of seven I–Bi–Bi–I layers, as shown in Supplementary Fig. 7. Unlike other Bi-rich bismuth iodides such as Bi_4_I_4_ with 1D structure^30^, the BiI we obtained is a member of 2D family and could be used to build the blocks of van der Waals heterostructures with other 2D atomic crystals. Moreover, considering the versatility of 1D Bi_4_I_4_ in thermoelectric, topological insulator, and superconductivity^31^, the 2D BiI may also exhibit similar properties.

### Dual bandgap of Bi_x_I_y_

**Fig. 3:**
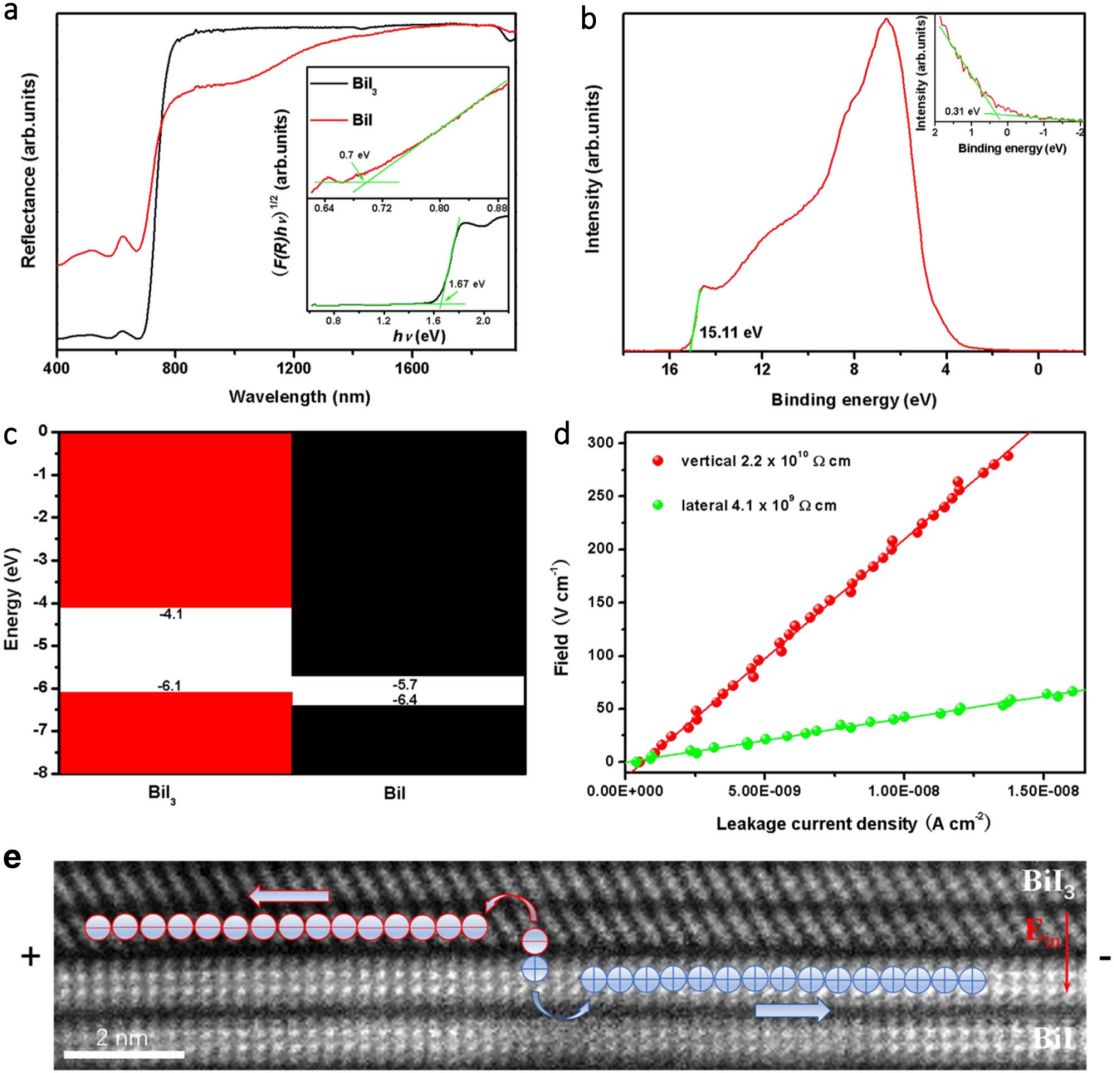
Band structure and charge separation kinetics of Bi_x_I_y_. **a** Diffuse reflectance spectra of commercial BiI_3_ and Bi_x_I_y_. Inset: calculated optical bandgaps of BiI_3_ layer and BiI layer in the Bi_x_I_y_ using the Tauc method by assuming an indirect bandgap. As using the reflectance, the Tauc relation is: (*F*(*R*)*hυ*)^1/2^ = *A*(*hυ-E*_g_), where *A* is a constant, *h* the Planck constant, *υ* the photon frequency, *F*(*R*) = (*1* *−* *R*)^2^/2 *R*, *R* the reflectance. The linear extrapolation (the green lines) in the absorption edge region of (*F*(*R*)*hυ*)^1/2^ versus *hυ* curve is used to determine the bandgap *E*_g_. **b** UPS spectrum of Bi_x_I_y_, the linear extrapolation (the green line) is used to determine the cutoff energy (15.11 eV). Inset: linear extrapolation (the green lines) in the low-binding-energy region, the energy (0.31 eV) determined is used to calculate the valence band energy (*E*_*v*_) as: *E*_*v*_ = 21.22 – 15.11 + 0.31 = 6.42 eV of BiI layer in the Bi_x_I_y_. **c** Band diagram of Bi_x_I_y_ exhibits a dual bandgap, the values of *E*_c_ (conduction band energy) and *E*_v_ (valence band energy) of BiI_3_ are extracted from ref. ^32^. **d** A typical leakage current-field relation of the Ag/Bi_x_I_y_/Ag devices tested by probe, Ag paste was used to fabricate the device, the red and green symbols are experimental data, the solid lines are linear fitting of the experimental data. **e** Excitation of electron–hole pairs separated at the BiI_3_–BiI interface. The cross-sectional image is cut out from a STEM image and used as a schematic diagram. The red and blue circles represent electron and hole, respectively. The red and blue arrows illustrate their flow under bias.

The bandgap of the Bi_x_I_y_ was measured by UV–Vis–NIR diffuse reflectance spectroscopy (DRS) and shown in Fig. 3a. The reflectance of Bi_x_I_y_ exhibits an indirect band nature with a sharp increase at 670–810 nm, assigned to the thick BiI_3_ layers, and a gentle rise after 810 nm, assigned to BiI layers. Namely, Bi_x_I_y_ exhibits a dual bandgap. The dual bandgap of Bi_x_I_y_ is also confirmed by the absorption spectrum (Supplementary Fig. 8) of a typical grown Bi_x_I_y_. Based on the DRS result, the bandgap energy (E_g_) of BiI layer was obtained by the Tauc method with 0.70 eV, dramatically lower than that of BiI_3_ (1.67 eV) layer. The reduced bandgap of BiI promotes photo-responses up to 1800 nm in the Bi_x_I_y_, as shown in Supplementary Fig. 9.

Figure 3b shows ultraviolet photoelectron spectroscopy (UPS) of the Bi_x_I_y_, which exhibits different cutoff energy (15.11 eV) and the energy (0.31 eV) extracted from linear extrapolation in the low-binding-energy region from which of BiI_3_ (about 16.75 eV and 1.25 eV, respectively, see ref. ^32^ and Supplementary Fig. 4). As seen from Fig. 2d, the distance between BiI_3_ and BiI layer (0.37 nm) is larger than which between BiI_3_ layers (0.33 nm), resulting in rich of BiI in the stripped surface of Bi_x_I_y_. Therefore, the valence band energy (*E*_*v*_) obtained by *E*_*v*_ = 21.22 – 15.11 + 0.31 = 6.42 eV is assigned to the BiI layer in the Bi_x_I_y_. The conduction band energy (*E*_*c*_) of BiI layer is then calculated by *E*_*c*_ = *E*_*v*_ + *E*_*g*_ = 5.72 eV. The band diagram of Bi_x_I_y_ heterostructure with a dual-bandgap can be plotted using the above data, as shown in Fig. 3c. Bi_x_I_y_ exhibits large and anisotropy resistivities of 4.1 × 10^9^ Ω cm for lateral (**E**⊥c) and 2.2 × 10^10^ Ω cm for vertical direction (**E**∥c), respectively, as shown in Fig. 3d. The resistivity of Bi_x_I_y_ is comparable to that of BiI_3_ (10^8^−10^13^ Ω cm)^12–25^, indicating a negligible effect of BiI layer with small bandgap (0.7 eV) to the resistivity of Bi_x_I_y_ due to its few numbers.

It can be deduced from the band diagram that electron injection from BiI_3_ layer to BiI layer would happen at the BiI_3_–BiI interface, which is confirmed by the transient absorption (TA) measurement (Supplementary Fig. 10). As seen from Supplementary Fig. 10, BiI_3_ exhibits an absorption bleaching at 645–675 nm, but no bleaching was observed in the Bi_x_I_y_. Photocarriers generated by incident laser can form full-filled electrons in the conduction band of BiI_3_, resulting in filled state bleaching. However, the bleaching disappeared in the Bi_x_I_y_ due to photoexcited electron transferring from BiI_3_ to BiI layers at the BiI_3_-BiI interface instead of maintaining a filled state in the conduction band of BiI_3_. The electron injection is also confirmed by a much weaker fluorescence around 700 nm of Bi_x_I_y_ than BiI_3_, as shown in Supplementary Fig. 11. Due to the electron injection from BiI_3_ layer to BiI layer, a built-in field with a direction from BiI_3_ to BiI emerges at the BiI_3_-BiI interface, which would prompt the electron–hole separation under X-ray radiation, as shown in Fig. 3e. The separation of electrons and holes into different layers would weaken their recombination and promote charge collection.

### X-ray response with low detection limit

As mentioned above, the heterostructure of Bi_x_I_y_ has high resistivity and shows benefits for charge separation. Moreover, Bi bilayer is observed in the middle of I–Bi–Bi–I four atomic layers (Supplementary Fig. 6) and further confirmed by the Raman spectrum of bismuth (Supplementary Fig. 12), which is promising for ultrafast electron transport because of the ultrahigh electron mobility of bismuth^33^. Therefore, Bi_x_I_y_ is expected to have a good X-ray response with low noise.

**Fig. 4:**
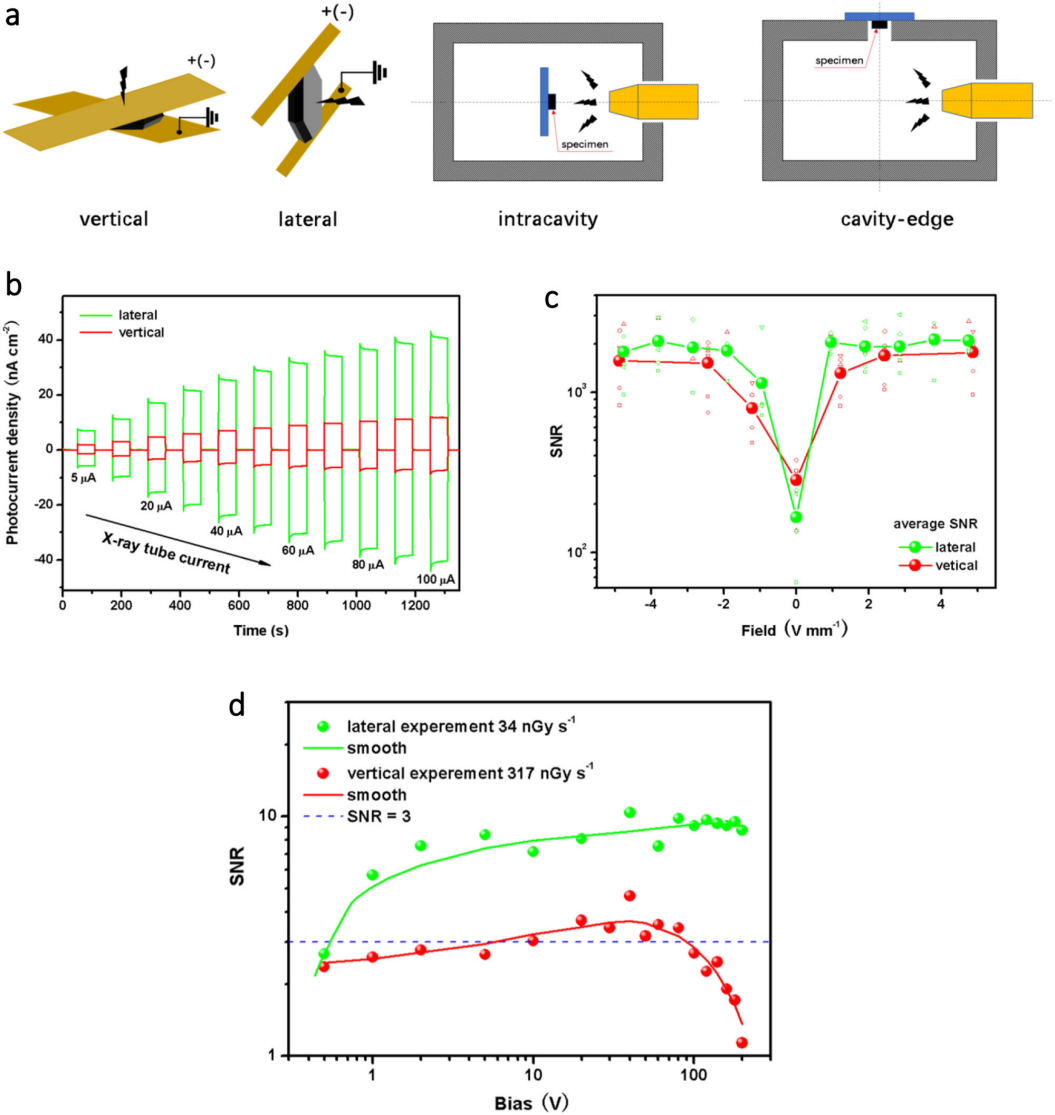
Room temperature device performance. **a** Illustration of X-ray detector (the Cu tapes cover the whole surface for full charge collection) and measurement configuration. **b** Anisotropic on/off X-ray responses of Bi_x_I_y_ at different dose rates measured by intracavity configuration under 1 V mm^−1^ bias. **c** Anisotropic bias-dependent SNR (average from values under different dose rates with an X-ray tube current of 5–100 μA) measured by intracavity configuration. **d** Anisotropic bias-dependent SNR measured by cavity-edge configuration. The blue dotted line represents a SNR of 3, so the detection limits are 34 nGy s^−1^ for lateral and 317 nGy s^−1^ for vertical devices, respectively. The solid lines are smooth of the experimental data.

Bulk (2.1 × 2.1 × 0.4 mm^3^) X-ray detectors were fabricated with a device structure of Cu/ Bi_x_I_y_/Cu. The copper conductive tapes (0.06 mm thickness) were pasted on both sides of the surfaces parallel or perpendicular to (001) to fabricate the vertical or lateral devices, as shown in Fig. 4a. The devices were exposed to a source with X-ray photon energy up to 70 keV. Then X-ray induced photocurrents were measured by a normal intracavity direct radiation configuration and a cavity-edge leakage radiation configuration, as shown in Fig. 4a.

As seen from Fig. 4b, the photocurrents of lateral and vertical devices measured by intracavity configuration under various X-ray dose rates reveal clear on/off responses with anisotropy. A detailed on/off response of Bi_x_I_y_ (Supplementary Fig. 13) shows rise/fall times of 49/71 ms for lateral device and 74/98 ms for the vertical device under 1 V mm^−1^ bias, which is comparable to the single crystal BiI_3_ (110/120 ms) grown by physical vapor transport (PVT)^14^ and the printable MAPbI_3_ device (less than 50 ms) for imaging^34^. The signal-to-noise ratios (SNR) calculated from the on/off responses (method described in ref. ^26^.) show stable and large average values around 2000 of the lateral device and 1600 of the vertical device, under various X-ray dose rates and bias larger than 1 V mm^−1^, as shown in Fig. 4c.

We also performed a leakage X-ray radiation measurement using a cavity-edge configuration (Fig. 4a) to examine the responses of the Bi_x_I_y_ detector in a simulated radiation leakage environment. As seen in Supplementary Fig. 14, the photocurrents induced by small leakage radiations still exhibit clear on/off responses in lateral and vertical devices. According to IUPAC standard, the dose rate with an SNR value of 3 is defined as the lowest detection limit at a given electric field. The lowest detection limit, representing the minimum X-ray dose rate used for inspection, is an important parameter relevant to health risk during X-ray security examinations or X-ray medical inspections^1,2^. As seen from Fig. 4d, the lowest detection limit of the lateral device achieved a very small value of 34 nGy s^−1^, which is comparable to the excellent X-ray detectors with low detection limit^4–8^. The detection limit of the vertical device also achieves a small value of 317 nGy s^−1^, much lower than that required for regular medical diagnostics (5.5 μGy s^–1^)^35^.

### X-ray sensitivity and stability

**Fig. 5:**
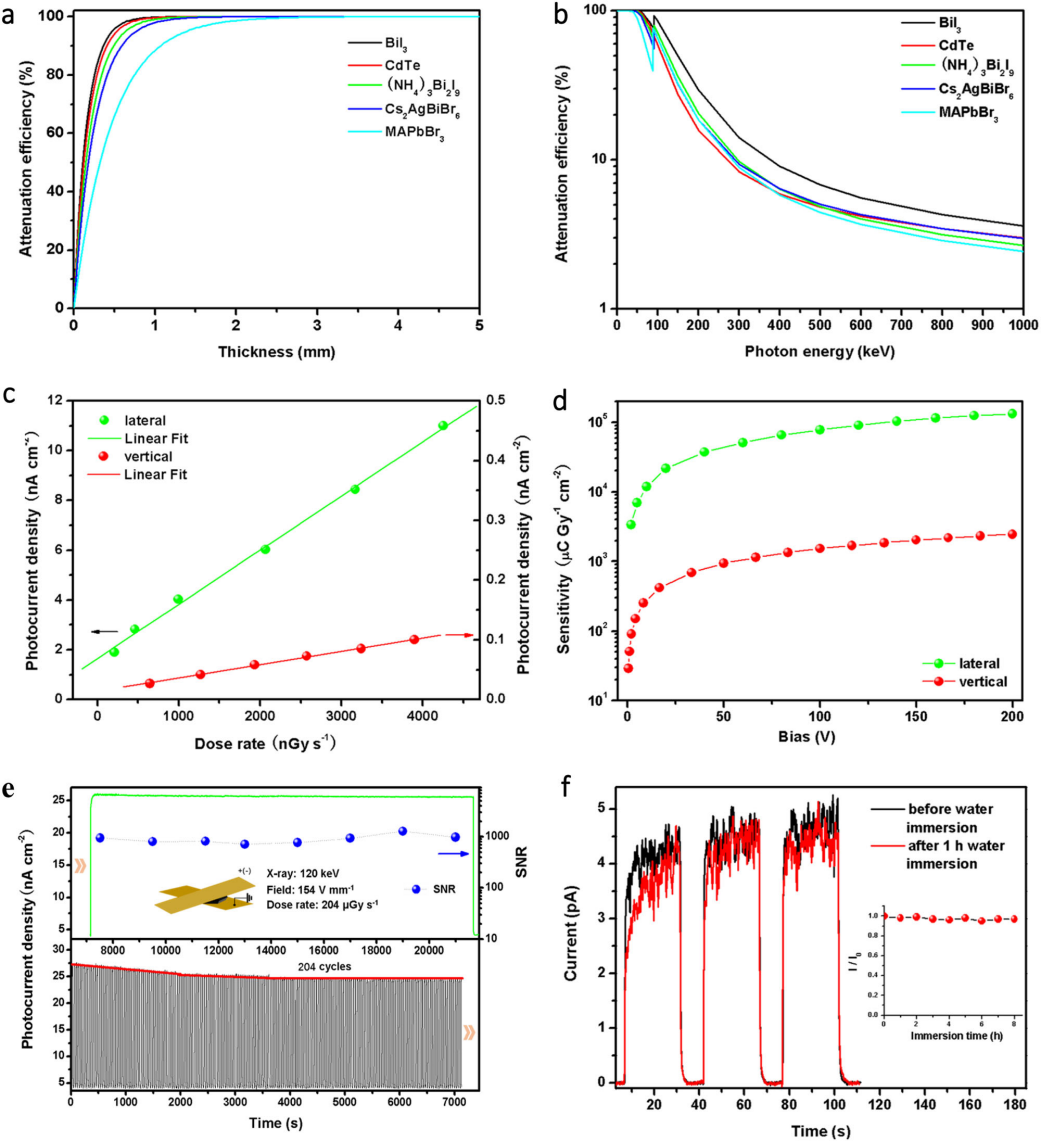
X-ray attenuation, sensitivity, and stability of Bi_x_I_y_ detector at room temperature. **a**, **b** Attenuation efficiencies of BiI_3_, (NH_4_)_3_Bi_2_I_9_, Cs_2_AgBiBr_6_, MAPbBr_3_ and CdTe semiconductors versus thickness to 50 keV X-ray photons (**a**) and photon energy (**b**). The curves were calculated by employing the NIST XCOM photon cross-section database^43^. **c** Anisotropic X-ray photocurrent densities at different dose rates measured by cavity-edge configuration under 1 V mm^−1^ bias, the linear fittings (solid lines) are used to calculate the sensitivity. **d** Anisotropic X-ray sensitivities at different bias measured by cavity-edge configuration. **e** Device stability under repeated and continuous X-ray radiation. The black line is 204 repeated on/off response, followed by a response (green line) with a long continuous “on” time. The red line illustrates a stable “on” current is achieved after a repeated on/off process. The blue symbols illustrate the fluctuation of device SNR during the long-time continuous X-ray radiation. Inset: a Cu/Bi_x_I_y_/Cu vertical device used for radiation stability test under 120 keV/204 uGy s^−1^ X-ray radiation and 154 V mm^−1^ field. **f** Device stability under humidity. The specimens, after water immersion, were dried by handkerchief tissues first and then repasted the Cu tapes for the following I–V tests. Inset: the X-ray response current changed with water immersion time up to 8 h, *I*_0_ is the response current without water immersion. The absorption of the copper conductive tape was subtracted from all the data shown in (**a**–**f**).

As mentioned above, thick BiI_3_ layers constitute the main body of Bi_x_I_y_ with good X-ray radiation attenuation efficiency attributed to its high atomic number (Z_Bi_ = 83, Z_I_ = 53), and high density (5.8 g cm^−3^). As seen from Fig. 5a, b, BiI_3_ showed a much better X-ray attenuation efficiency than MAPbBr_3_. For 50 keV hard X-ray, BiI_3_ would attenuate 99.82% of the incident photons, while MAPbBr_3_ 88.41% at 1 mm thickness. Therefore, high attenuation efficiency enables Bi_x_I_y_ to adequately absorb X-ray with reduced thickness, accelerating the charge collection. The charge collection ability of Bi_x_I_y_ characterized by a *μτ* product, where μ is the carrier mobility and τ the carrier lifetime, is derived by fitting the photoconductivity using Hecht equation^36^, as shown in Supplementary Fig. 15. The Bi_x_I_y_ exhibits anisotropic *μτ* products of 3.0 × 10^−3^ cm^2^ V^−1^ (lateral) and 4.4 × 10^−5^ cm^2^ V^−1^ (vertical) respectively. The corresponding lateral *μτ* products is comparable to that of perovskites with good X-ray detection properties^5,26,37^. The mobilities of Bi_x_I_y_ measured by the space charge-limited current (SCLC) method confirmed the strong anisotropic charge transport with values of 53 cm^2^ V^−1^ s^−1^ (lateral) and 0.15 cm^2^ V^−1^ s^−1^ (vertical), respectively, as shown in Supplementary Fig. 16. According to the electronic dimensionality theory^38^, the electronic bands are more dispersive in the (001) plane of Bi_x_I_y_ owing to the strong in-plane chemical bonds interaction, while more localized perpendicular to the (001) plane induced by the weak out-of-plane van der Waals interaction. Therefore, Bi_x_I_y_ shows a better charge collection ability in the lateral direction.

Strong X-ray attenuation, high resistivity, good charge collection ability, and the resulting apparent responses at weak radiation indicate the Bi_x_I_y_ is highly sensitive to X-ray. The sensitivity of X-ray detectors is derived from the current-dose rate relations, as shown in Fig. 5c. The lateral device has much larger sensitivity than the vertical device, as shown in Fig. 5d. The obtained sensitivity of lateral device achieved a high value of 4.3 × 10^4^ μC Gy^−1^ cm^−2^ at 24 V mm^−1^ (50 V) bias, which is comparable to the newly reported high-sensitive detectors^37,39–42^, shown in Supplementary Fig. 17. Moreover, the sensitivity of lateral device achieves nearly one order of magnitude higher than that of the lateral BiI_3_ single crystal detector (0.5 × 10^4^ μC Gy^−1^ cm^−2^ at 20 V mm^−1^)^19^, confirmed the advantage of heterostructure of Bi_x_I_y_ for X-ray detection. However, the sensitivity of vertical device (253 μC Gy^−1^ cm^−2^ at 19.5 V mm^−1^) is smaller than which of the vertical BiI_3_ single crystal detector (660 μC Gy^−1^ cm^−2^ at 20 V mm^−1^)^19^. A larger distance between BiI_3_ and BiI layers than which between BiI_3_ layers leads to easier mechanical exfoliation of Bi_x_I_y_, however, harms the charge transport and then reduce the sensitivity of the vertical detector.

The Bi_x_I_y_ detector was exposed to repeated and continuous 120 keV X-ray (used for CT) with a dose rate of 204 μGy s^−1^ to evaluate the anti-radiation stability. As seen from Fig. 5e, Stable X-ray photocurrent with a high SNR of around 1000 was observed after 204 circles repeated radiation and followed continuous radiation more than 4 h, confirms the highly stability of Bi_x_I_y_ detector under high energy X-ray radiation. Moreover, nearly unchanged photocurrent intensity (Fig. 5f) of the Bi_x_I_y_ detectors could be observed even after 8 h water (20 °C) immersion (Supplementary Fig. 18), confirms its highly environmental stability. High-sensitive and high-stable X-ray response of Bi_x_I_y_ detector offers its great prospects in real applications.

## Discussion

In summary, we developed a handy and scalable solution method to first grow the macrosize van der Waals heterostructure of Bi_x_I_y_ with regular shapes consisting of adjacent thick BiI_3_ (main) and thin BiI (minor) 2D layers. The Bi_x_I_y_ heterostructure X-ray detectors exhibit stable response and anisotropic properties at different crystal orientation. The lateral device realized a high sensitivity of 4.3 × 10^4^ μC Gy^−1^ cm^−2^ with a very low detection limit of 34 nGy s^−1^, meeting the demands of medical inspection to reduce the X-ray exposure to the human body. On the other hand, the Bi_x_I_y_ photodetectors are versatile and present a photo response ranging up to 1800 nm, revealing its potential for near-infrared detection. Generally speaking, our results inspire the exploration of van der Waals heterostructure materials for high-sensitive X-ray detection.

## Methods

### Precursor solution preparation

All the purchased chemical reagents except Au (99.999%) were of analytical reagent grade purity and used without further purification. Solution 1 was prepared by 5.5 g Bi_2_O_3_ it was dissolved in 20 ml 55% hydroiodic acid at room temperature. Solution 2 was prepared by 2 g Au and 10 g I_2_. They were dissolved in 5 ml 55% hydroiodic acid for 3 days at room temperature. Solution 3 was prepared by 10% hydroiodic acid mixed with ethanol in a volume ratio of 1:1. solution 1 and solution 2 were mixed and diluted by solution 3 to 40 ml. The prepared 40 ml diluted solution was used as a precursor solution.

### Solution pretreatment and refinement

The pretreatment and refinement procedures are schematically illustrated in Fig. 1a. Briefly, the obtained precursor solution was put into a 50 ml Ф20-mm conical flask. The flask was placed in a sealed beaker with 100 ml 1, 4-butyrolactone (GBL) for solvothermal treatment. Three times treatments are needed. The solvothermal treatment was performed in an 80 °C oven. After the first-time treatment (3–5 days), the solution was concentrated to 35 ml. The concentrated solution was diluted by solution 3 to 40 ml again for the second time solvothermal treatment. After the second time treatment, the solution was concentrated to 30 ml. The second time concentrated solution was diluted by ethanol to 35 ml for the third time solvothermal treatment. After the third time treatment, the solution was concentrated to 25 ml. The third time concentrated solution was then refined by hydrothermal treatment in a 60 °C oven for 3 days. Some small bulks with an irregular shape formed at the bottom of the conical flask after hydrothermal. The upper portion of the supernatant was then carefully transferred into another clear container to grow high-quality crystals.

### Crystal growth

The solution, after pretreatment and refinement, was then handled by water bath (Fig. 1a) at room temperature to grow crystals. More than 7 days of growth without disturbance is needed to obtain millimeter crystals. The obtained crystals were washed by ethanol one time and dichloromethane two times followed. Bulks after washing dried naturally in the air and used for the following material characterization, device preparation, and test.

### Characterization

Powder X-ray diffraction was performed on a D8-DISCOVER diffractometer with Cu Kα (λ = 1.542 Å) radiation. The X-ray Photoelectron Spectroscopy (XPS) and Ultraviolet Photoelectron Spectroscopy (UPS) were performed on a Thermo Fisher ESCALAB XI + photoelectron spectrometer. Freshly exfoliated surface after 30 s Ar ion sputtering was used for XPS and UPS measurements. Thermogravimetric analysis (TGA) was carried out under continuous nitrogen flow using a NETZSCH STA 449F3 thermal gravimetric analyzer. The sample was held on a platinum pan, and heated at a rate of 5 °C min^−1^ up to 600 °C. AFM measurements were carried out in an Oxford Instruments Asylum Research Cypher S atomic force microscope with a contact mode. An IT500 scanning electron microscope (SEM) with a maximum 30 kV electron beam accelerating voltage was employed to observe the surface morphology of Bi_x_I_y_. STEM observations of the cross-section specimens were carried out in an aberration-corrected STEM microscope (Titan G2 60-300, Thermofisher equipped with a field emission gun) with 300 kV electron beam accelerating voltage. The probe convergence angle was 24.5 mrad, and the angular range of the HAADF detector was from 79.5 to 200 mrad. The cross-sectional TEM specimens were prepared by a dual-beam focused ion beam (FIB) nanofabrication platform (Helios 600i, Thermofisher). The UV–Vis–NIR diffuse reflectance spectroscopy (DRS) was measured by a HATACHI UH4150 spectrometer over the spectral range of 360−2000 nm. Room temperature photoluminescence and Raman spectra were collected by a Horiba LabRam HR Evolution microscopic confocal Raman spectrometer using a 6.8 mW, 532 nm CW Nd: YAG laser as an excitation source. The laser beam was focused to a spot size of about 0.7 μm in diameter. Transient absorption (TA) measurements were performed on a HARPIA-TA system (Light Conversion) at room temperature. A 1030 nm pulsed laser with 100 kHz repetition rate and 190 fs pulse duration was divided into two beams to generate pump laser and probe light, respectively. The pump laser of 480 nm was generated from an optical parametric amplifier system (OPA, Light Conversion) pumped by one beam of 1030 nm laser. The probe light was generated by exciting a sapphire plate by another beam of 1030 nm laser.

### Photo and X-ray response measurement

The device for photodetection was fabricated on the (001) surface of a Bi_x_I_y_ bulk, as shown in Supplementary Fig. 10. A pair of Ag electrodes with an interval of 0.5 mm was formed by painting Ag paste on a freshly exfoliated surface and then dried at 100 °C in the air. The area between Ag electrodes formed the light absorption area of the photodetector. The I–V characteristic under ambient light and infrared irradiation was measured by a KEITHLEY 2450 source meter. A YSL SC-PRO 7 supercontinuum source was used to generate CW infrared laser. Devices with Cu tape pasted on a pair of adjacent (100) or (001) surfaces formed the Cu/ Bi_x_I_y_/Cu structure (Fig. 3a), and were used for X-ray detection measurements. The X-ray detection performance was measured in a Pb cavity for intracavity mode and in a Ф 5  mm hole on the side of the cavity with a light-proof cover for cavity-edge mode, as shown in Supplementary Fig. 13. A commercially available MOXTEX MagPro Mini-X tube with a tungsten target and 12 W maximum power output was used as the X-ray source. The X-ray tube was operated with a constant 50 kV voltage. The total X-ray dose was modulated by changing the current of the X-ray tube. The radiation dose rate was calibrated using a Radical ion chamber dosimeter. The X-ray photocurrent was measured by a KEITHLEY 2636B source meter. For the anti-radiation test, a 150 kV HAMAMATSU/L12161-07 microfocus X-ray source with 75 W maximum power was used.

## Supplementary information

Supplementary Information

Peer Review File

## Data Availability

Relevant data supporting the key findings of this study are available within the article, the Supplementary Information file and the Source Data file. All raw data generated during the current study are available from the corresponding authors upon request. Source data are provided with this paper.

## Source data

Source Data

## References

[1] Brenner DJ (2001). Estimated risks of radiation induced fatal cancer from pediatric CT. Am. J. Roentgenol..

[2] Polischuk BT (1998). Selenium direct converter structure for static and dynamic X-ray detection in medical imaging applications. Proc. Med. Imag..

[3] He YH (2022). Detecting ionizing radiation using halide perovskite semiconductors processed through solution and alternative methods. Nat. Photon.

[4] Song, Y. L. et al. Atomistic surface passivation of CH_3_NH_3_PbI_3_ perovskite single crystals for highly sensitive coplanar-structure X-ray detectors. *Research* **2020**, 5958243 (2020). 10.34133/2020/5958243 PMC7528034 33043296

[5] Sarah D (2021). High-sensitivity high-resolution X-ray imaging with soft-sintered metal halide perovskites. Nat. Electron.

[6] Zhang P (2022). Ultrasensitive and robust 120 keV hard X-ray imaging detector based on mixed-halide perovskite CsPbBr_3−n_ I_n_ single crystals. Adv. Mater..

[7] Zhuang RZ (2019). Highly sensitive X-ray detector made of layered perovskite-like (NH_4_)_3_Bi_2_I_9_ single crystal with anisotropic response. Nat. Photon..

[8] Liu Y (2021). Ligand assisted growth of perovskite single crystals with low defect density. Nat. Commun..

[9] Szeles C (2004). CdZnTe and CdTe materials for X-ray and gamma ray radiation detector applications. Phys. Status Solidi B.

[10] Li, J. et al. Synthesis of 2D layered BiI_3_ nanoplates, BiI_3_/WSe_2_ van der Waals heterostructures and their electronic, optoelectronic properties. *Small* **13**, 1701034 (2017). 10.1002/smll.201701034 28791794

[11] Polyakov A (2019). A bismuth triiodide monosheet on Bi_2_Se_3_ (0001). Sci. Rep..

[12] Nikolas JP (2013). Band gap and structure of single crystal BiI_3_: resolving discrepancies in literature. J. Appl. Phys..

[13] Saito T (2016). BiI_3_ single crystal for room-temperature gamma ray detectors. Nucl. Instrum. Methods Phys. Res. A.

[14] Sun H (2018). Preparation and characterization of free-standing BiI_3_ single-crystal flakes for X-ray detection application. J. Mater. Sci.: Mater. Electron..

[15] Gokhale SS (2015). Growth, fabrication, and testing of bismuth triiodide semiconductor radiation detectors. Radiat. Meas..

[16] Azaree T (2011). Characterization of bismuth tri-iodide single crystals for wide bandgap semiconductor radiation detectors. Nucl. Instrum. Methods Phys. Res. A.

[17] Aguiar I (2009). Bismuth tri-iodide polycrystalline films for X-ray direct and digital imagers. Nucl. Instrum. Methods Phys. Res. A.

[18] Fornaro L (2004). Bismuth tri-iodide polycrystalline films for digital X-ray radiography applications. IEEE Trans. Nucl. Sci..

[19] Liu YZ (2019). Electrical properties of X-ray detector based on bismuth tri-iodide single crystal with electrode configuration considering. Mater. Res. Express.

[20] Chaudhari R (2021). Bismuth tri-iodide-polystyrene composite for X-rays switching applications at room temperature. Radiat. Phys. Chem..

[21] Dmitriev Y (1999). Bismuth iodide crystals as a detector material: some optical and electrical properties. Proc. SPIE.

[22] Matsumoto M (2002). Bismuth tri-iodide crystals for nuclear radiation detectors. Trans. Nucl. Sci..

[23] Cho SB (2018). Intrinsic point defects and intergrowths in layered bismuth triiodide. Phys. Rev. Mater..

[24] Cuña A (2004). Growth of bismuth tri-iodide platelets by the physical vapor deposition method. Cryst. Res. Technol..

[25] Nason D (1995). The growth and crystallography of bismuth tri-iodide crystals grown by vapor transport. J. Cryst. Growth.

[26] Zhang YX (2020). Nucleation-controlled growth of superior lead-free perovskite Cs_3_Bi_2_I_9_ single-crystals for high-performance X-ray detection. Nat. Commun..

[27] Jain SM (2018). An effective approach of vapour assisted morphological tailoring for reducing metal defect sites in lead-free, (CH_3_NH_3_)_3_Bi_2_I_9_ bismuth based perovskite solar cells for improved performance and long-term stability. Nano Energy.

[28] Pennycook SJ, Jesson DE (1990). High-resolution incoherent imaging of crystals. Phys. Rev. Lett..

[29] Pennycook SJ, Jesson DE (1991). High-resolution Z-contrast imaging of crystals. Ultramicroscopy.

[30] Filatova TG (2007). Electronic structure, galvanomagnetic and magnetic properties of the bismuth subhalides Bi_4_I_4_ and Bi_4_Br_4_. J. Solid State Chem..

[31] Pisoni A (2017). Pressure effect and superconductivity in the β−Bi_4_I_4_ topological insulator. Phys. Rev. B.

[32] Lehner AJ (2015). Electronic structure and photovoltaic application of BiI_3_. Appl. Phys. Lett..

[33] Wang YW (2019). Engineering ultrafast charge transfer in bismuthene/perovskite nanohybrid. Nanoscale.

[34] Yong CK (2017). Printable organometallic perovskite enables large-area, low-dose X-ray imaging. Nature.

[35] Shearer DR (2000). Dose rate limitations of integrating survey meters for diagnostic X-ray surveys. Health Phys..

[36] Kasap SO (2000). X-ray sensitivity of photoconductors: application to stabilized a-Se. J. Phys. D..

[37] Pan WS (2017). Cs_2_AgBiBr_6_ single-crystal X-ray detectors with a low detection limit. Nat. Photon.

[38] Xiao ZW (2017). Searching for promising new perovskite-based photovoltaic absorbers: the importance of electronic dimensionality. Mater. Horiz..

[39] Li LQ (2019). Enhanced X-ray sensitivity of MAPbBr_3_ detector by tailoring the interface-states density. ACS Appl. Mater. Interfaces.

[40] Wei W (2017). Monolithic integration of hybrid perovskite single crystals with heterogenous substrate for highly sensitive X-ray imaging. Nat. Photon..

[41] Shen Y (2020). Centimeter-sized single crystal of two-dimensional halide perovskites incorporating straight-chain symmetric diammonium ion for X-ray detection. Angew. Chem..

[42] Zhang HJ (2020). High-sensitivity X-ray detectors based on solution-grown caesium lead bromide single crystals. J. Mater. Chem. C..

[43] Berger, M. J. et al. XCOM: photon cross sections database: NIST standard reference database 8 (NIST, 2013). https://www.nist.gov/pml/xcom-photon-cross-sections-database (2013).

