# Supplementary material for: Solution-grown BiI/BiI3 van der Waals heterostructures for sensitive X-ray detection
Source: Nat Commun. 2023 Mar 23;14:1621. doi: 10.1038/s41467-023-37297-z (PMC10036621; doi:10.1038/s41467-023-37297-z)
Supplement: Supplementary file 1 — Supplementary Information [file 41467_2023_37297_MOESM1_ESM.pdf]

# Supplementary Information

# Solution-grown BiI/$BiI_3$ van der Waals heterostructures for sensitive X-ray detection

Renzhong Zhuang[1, 2], Songhua Cai[3], Zengxia Mei[1], Huili Liang[1], Ningjiu Zhao[1], Haoran Mu[1], Wenzhi Yu[1, 4], Yan Jiang[1], Jian Yuan[1], Shuping Lau[3], Shiming Deng[5], Mingyue Han[1], Peng Jin[6], Cailin Wang[1], Guangyu Zhang[1, 4, *], Shenghuang Lin[1, *]

[1]Songshan Lake Materials Laboratory, Dongguan 523808, Guangdong, P. R. China.

[2]Fujian Provincial Key Laboratory of Welding Quality Intelligent Evaluation, Longyan University, Longyan, Fujian, P. R. China.

[3]Department of Applied Physics, The Hong Kong Polytechnic University, Hunghom, Kowloon, Hong Kong, P. R. China.

[4]Institute of Physics, Chinese Academy of Science, Beijing, 100190, P. R. China.

[5]HAMAMATSU Photonics (China) Co., LTD., Beijing, 100020, P. R. China.

[6]State Key Laboratory of Modern Optical Instrumentation, College of Optical Science and Engineering, Zhejiang University, Hangzhou, Zhejiang, China.

*Email Address: linshenghuang@sslab.org.cn (S. Lin) & gyzhang@sslab.org.cn (G. Zhang)

## Supplementary Figures

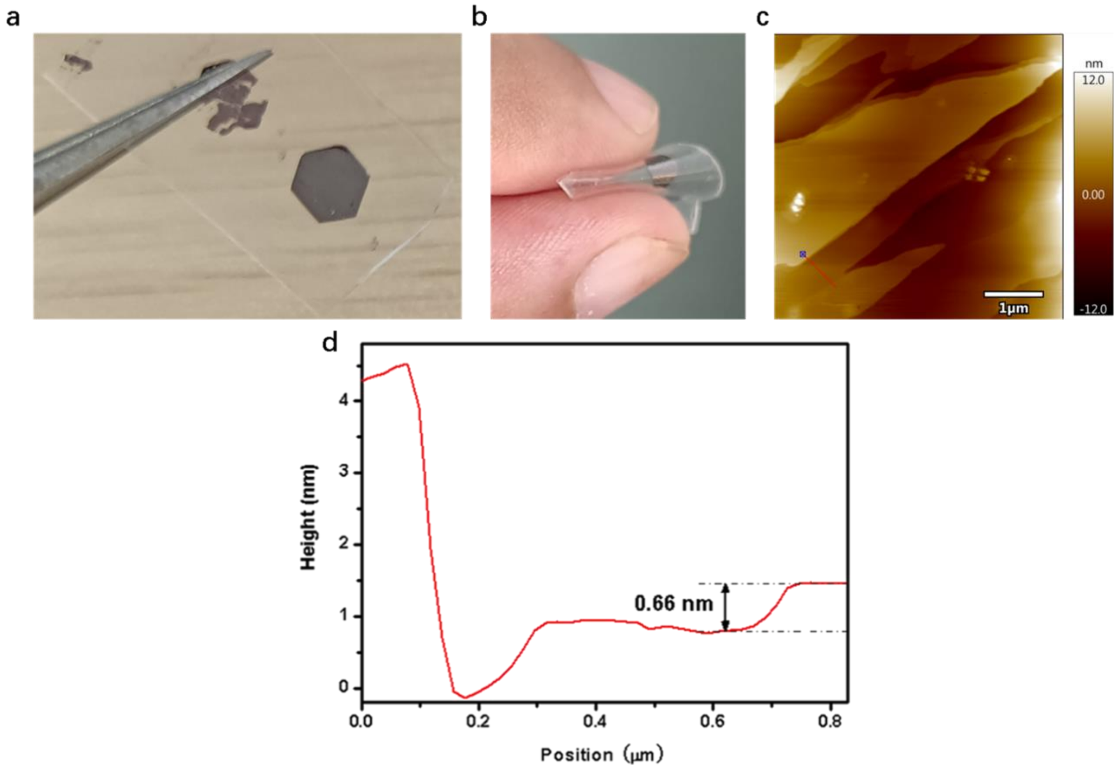


**Supplementary Figure 1 ｜Morphology characterization of $Bi_xI_y$. a**, **b**, Complete and flexible slices exfoliated by PDMS, respectively. **c**, **d**, AFM image (**c**) and corresponding surface profile (**d**) of a freshly exfoliated flake.

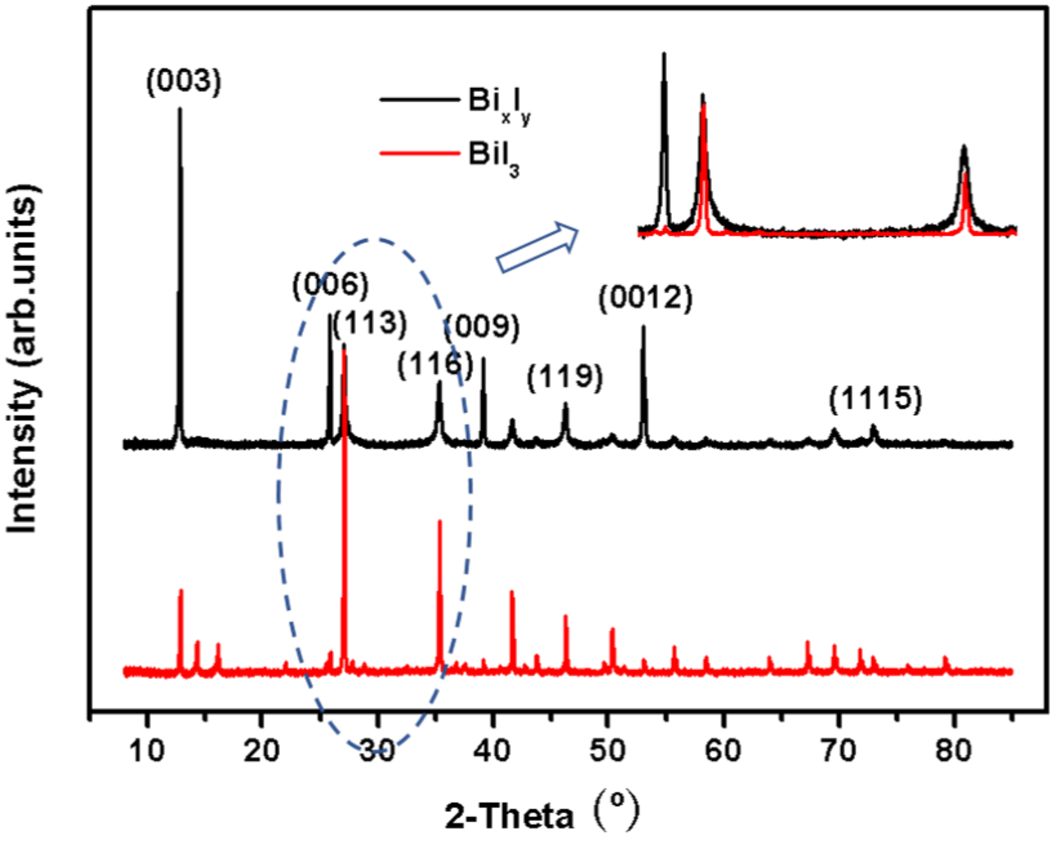


**Supplementary Figure 2 | Powder X-ray diffraction characterization**. XRD patterns of $Bi_xI_y$ and $BiI_3$ (Aladdin, 98%), the intensity of (113) peak is normalized in the insert to compare the peak width of $Bi_xI_y$ and $BiI_3$.

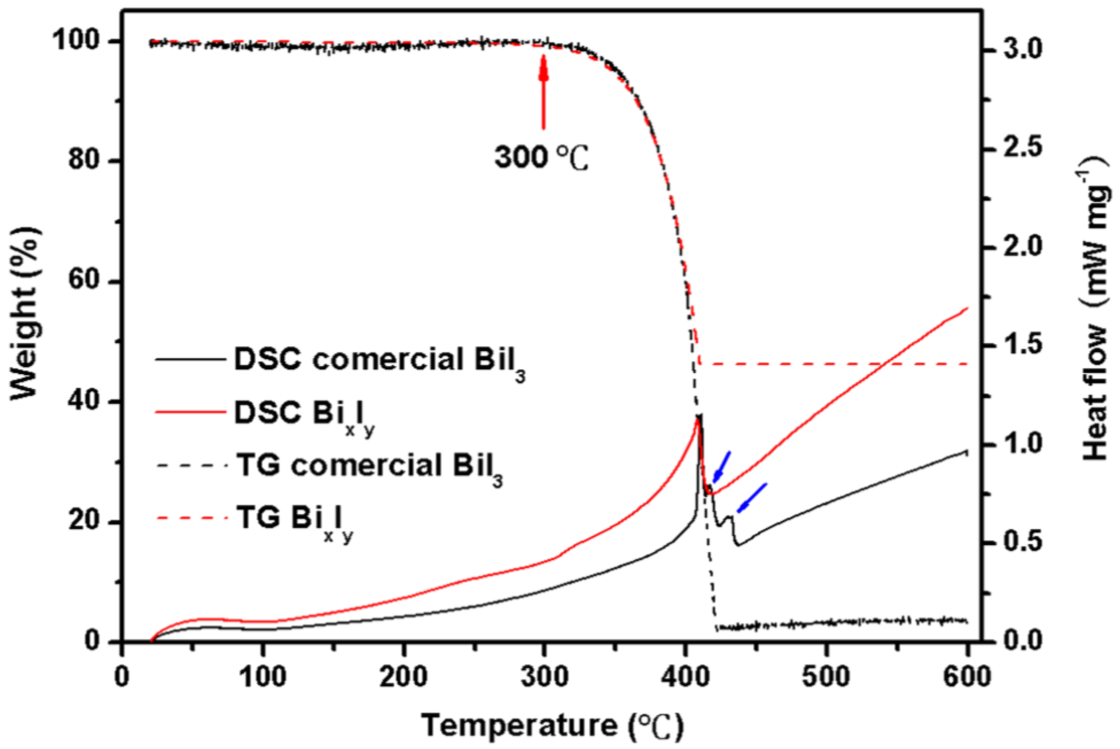


**Supplementary Figure 3 | Thermal analysis**. Thermogravimetric and differential scanning calorimetry analysis curves of $Bi_xI_y$ and commercial $BiI_3$. Two decomposition temperatures above melting point at 417 ℃ and 431 ℃ were observed in $BiI_3$, respectively.

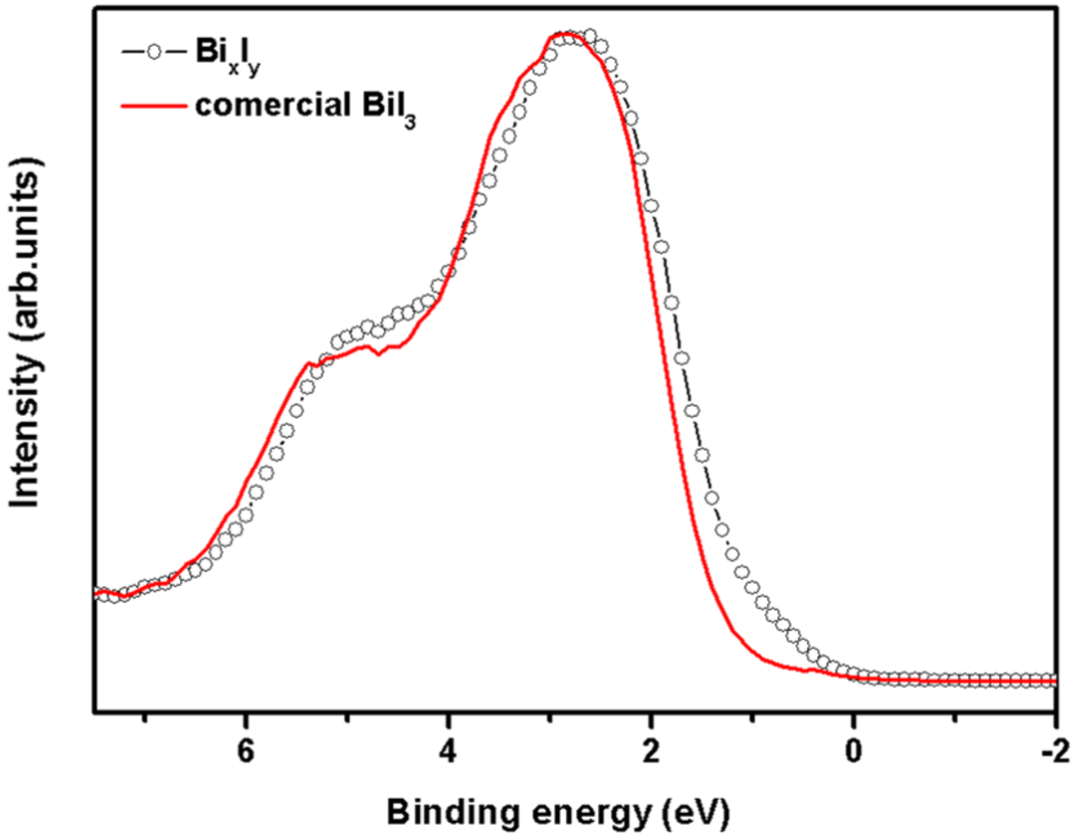


**Supplementary Figure 4 ｜Valance band XPS characterization.** XPS spectra of valance band BE of $Bi_xI_y$ and commercial $BiI_3$.

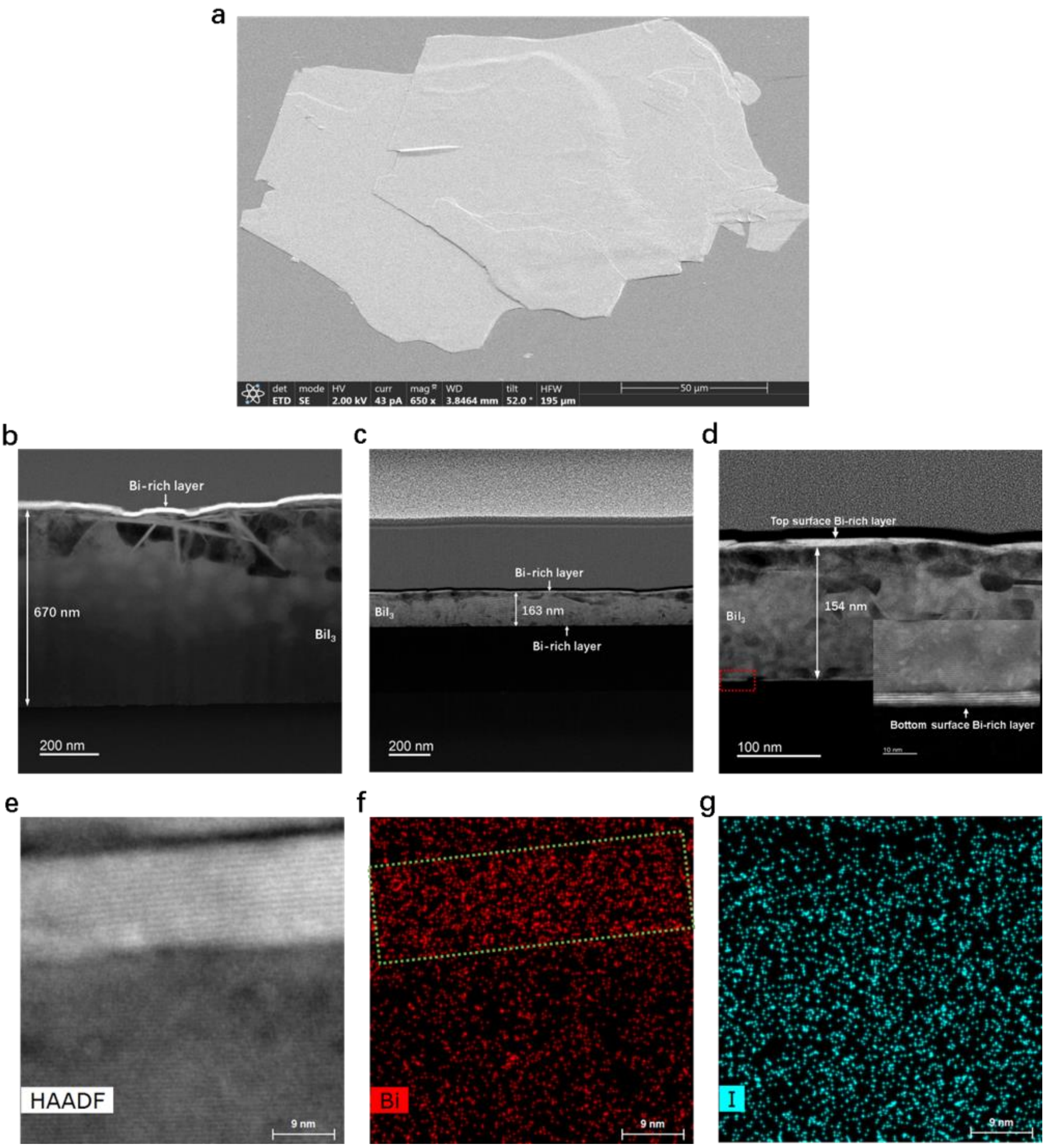


**Supplementary Figure 5 | Cross-sectional HAADF-STEM images of stripped $Bi_xI_y$ slices**. **a**, SEM image of $Bi_xI_y$ slices for FIB. **b-d**, Alternate stacking of thin bright Bi-rich layers and thick dark $BiI_3$ layers with 670 nm (**b**), 163 nm (**c**) and 154 nm (**d**) thickness. **e-g**, STEM-EDS mapping of Bi (**f**) and I (**g**) in a region with bright Bi-rich phase and dark $BiI_3$ (**e**).

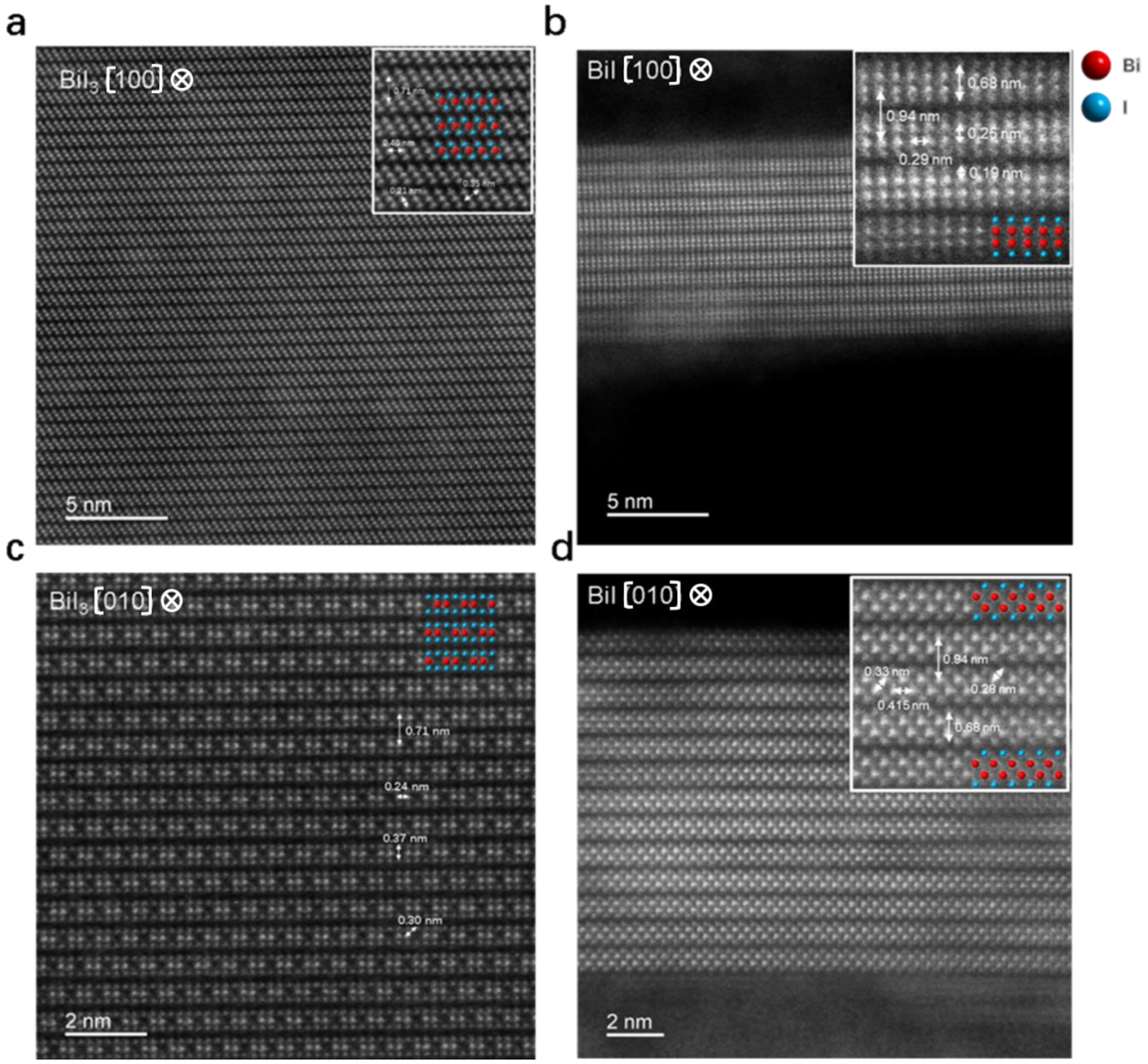


**Supplementary Figure 6 | Atomic arrangement of $BiI_3$ and BiI. a-d**, HAADF-STEM images of $BiI_3$ and BiI structure viewed along the [100] zone axis (**a**, **b**) and the [010] zone axis (**c**, **d**).

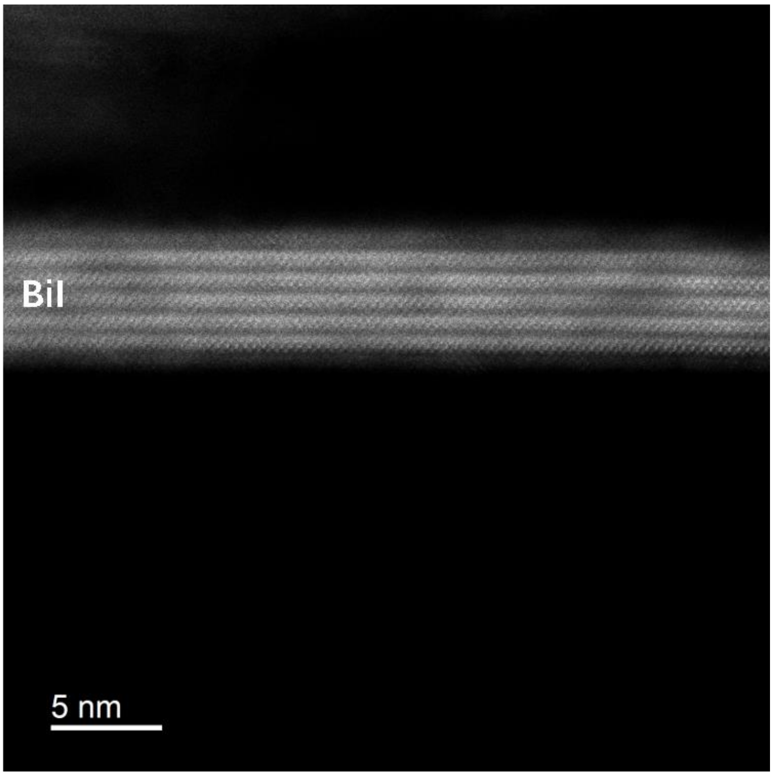


**Supplementary Figure 7 | Separated BiI layer**. HAADF-STEM image of an exfoliated BiI film with 7 I-Bi-Bi-I layers.

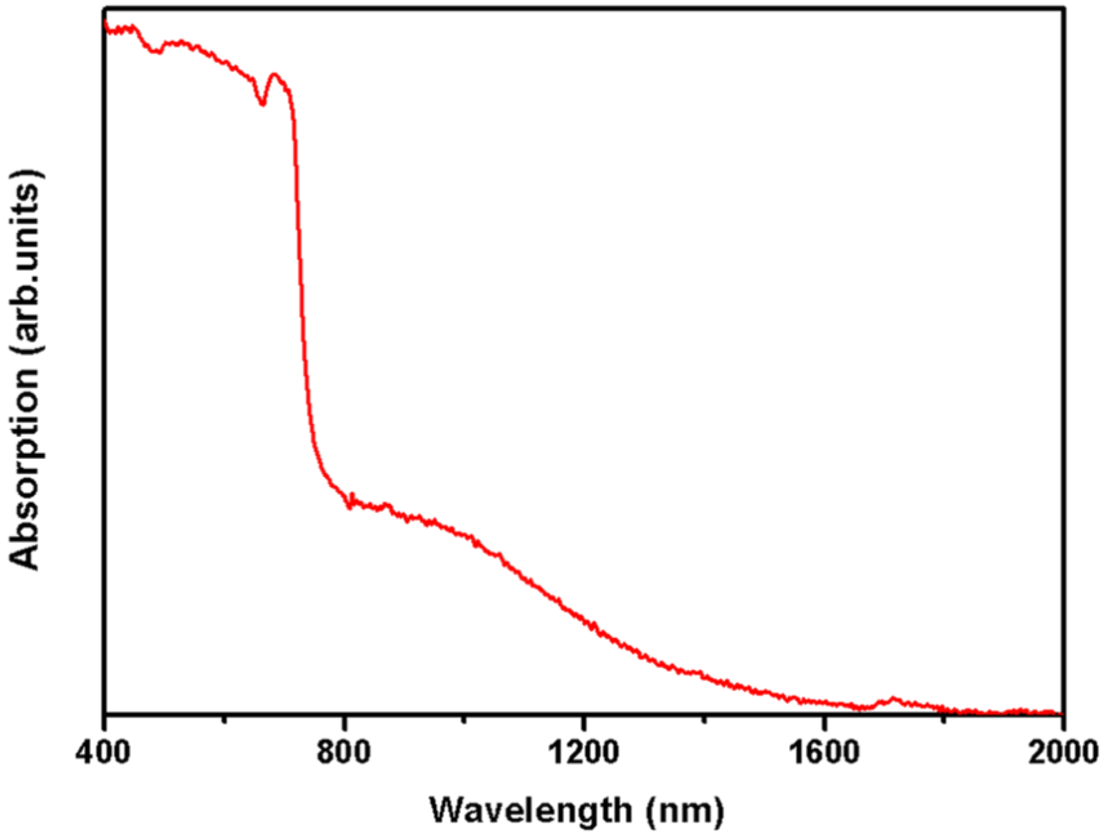


**Supplementary Figure 8 ｜ Optical absorption characterization.**

Absorption spectrum of $Bi_xI_y$ exhibits a dual bandgap.

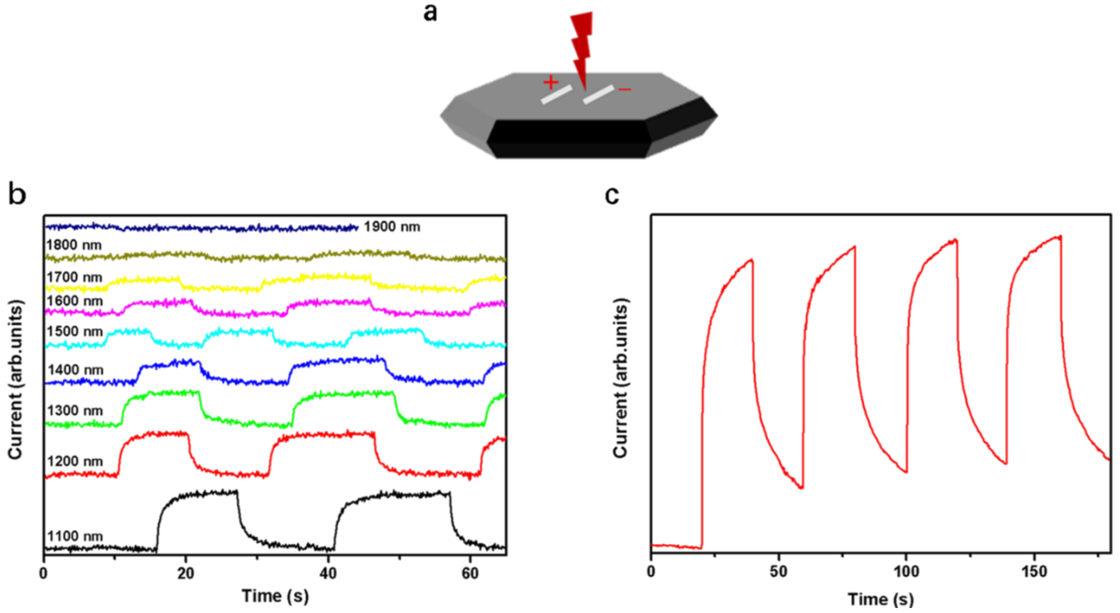


**Supplementary Figure 9 | photo response of the $Bi_xI_y$. a**, A planar-type Ag/$Bi_xI_y$/Ag photodetector made on the (001) surface of $Bi_xI_y$. **b**, Infrared on/off responses from 1100-1900 nm. **c**, On/off responses under ambient light.

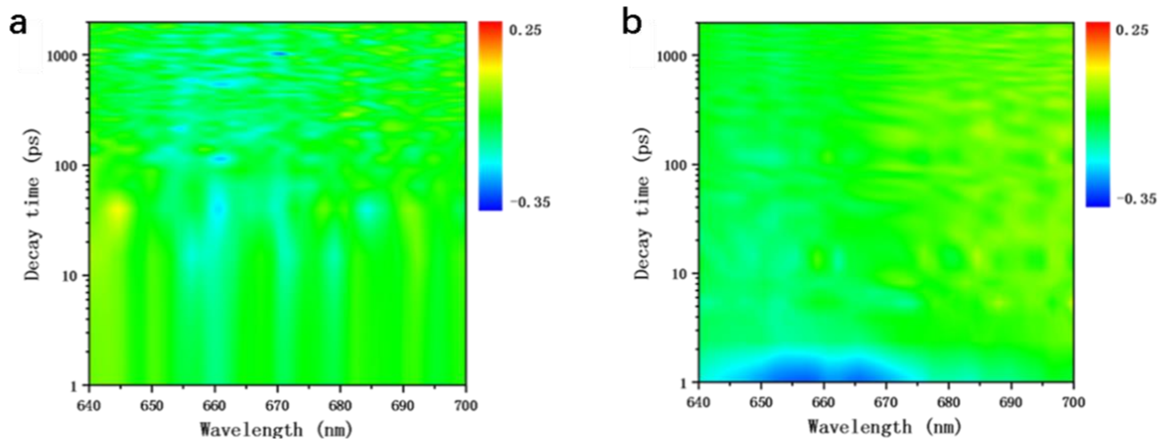


**Supplementary Figure 10 | Transient dynamics characterization. a, b,** Transient absorption spectra of $Bi_xI_y$ (**a**) and $BiI_3$ (film on a quartz plate prepared by a solution method described in supplementary ref. 1) (**b**).

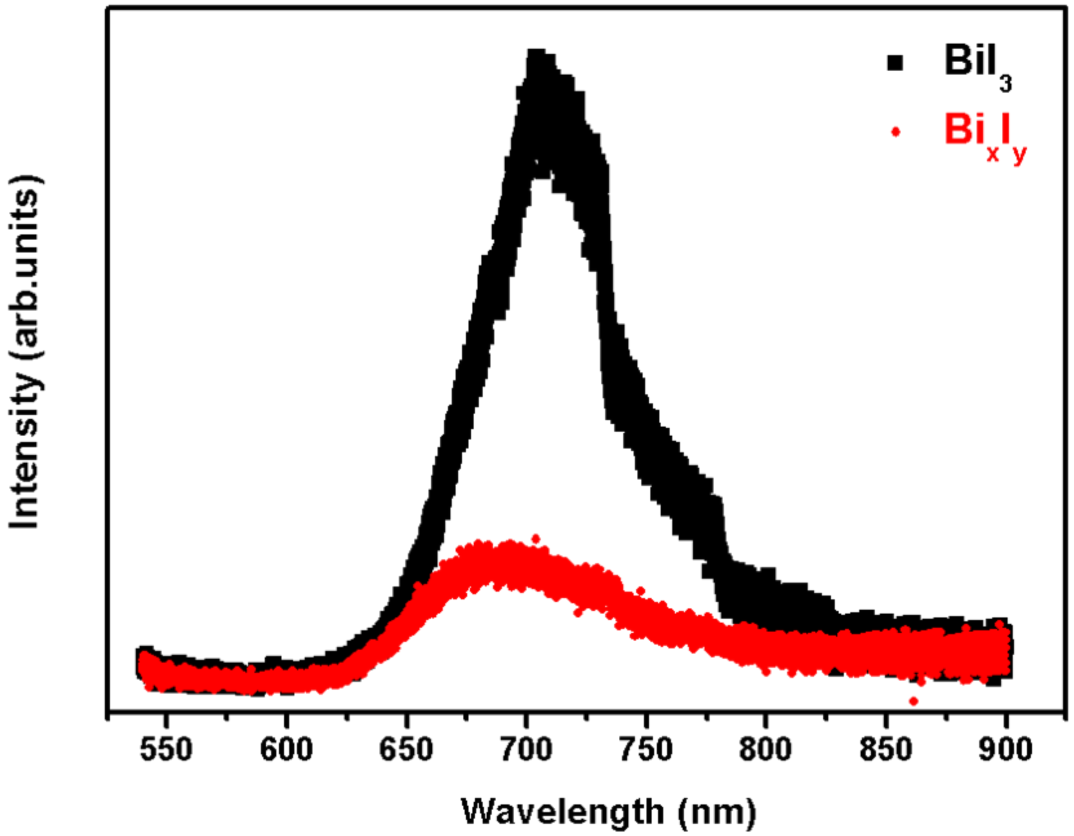


**Supplementary Figure 11 | Photoluminescence characterization.**

Photoluminescence spectrum of the $BiI_3$ and $Bi_xI_y$.

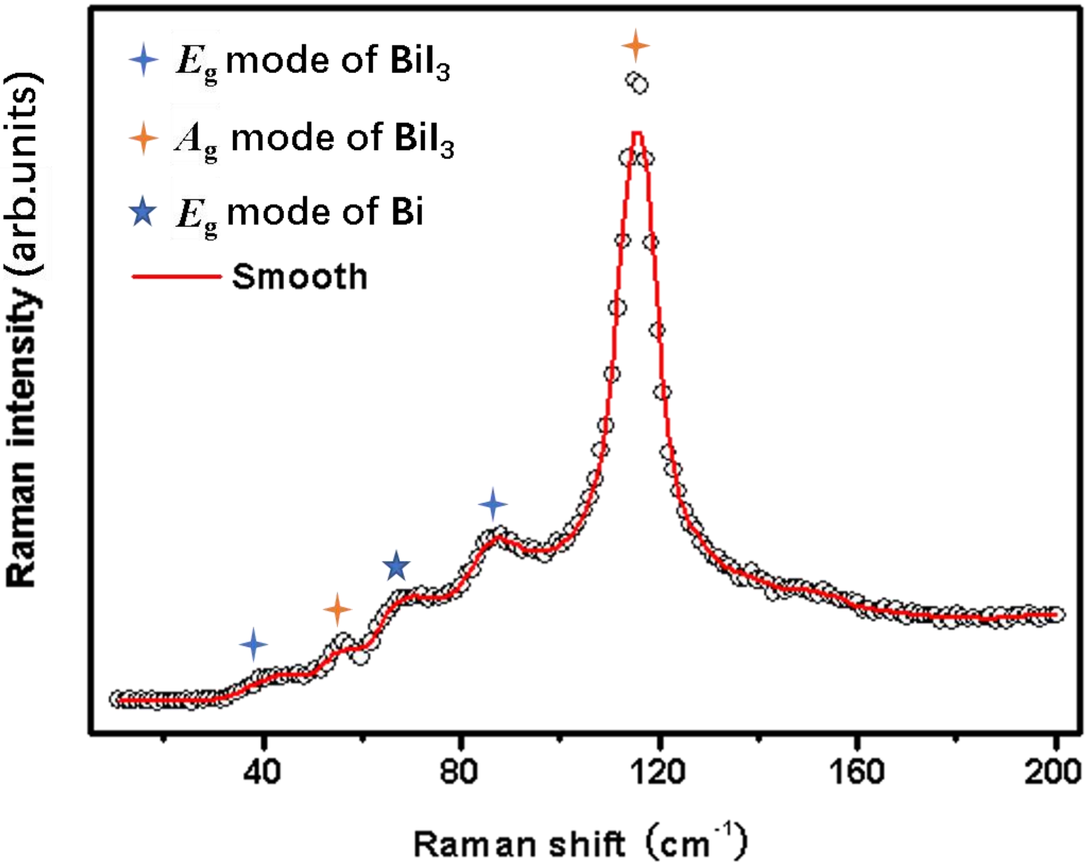


**Supplementary Figure 12 | Raman characterization.** Raman spectrum of the $Bi_xI_y$ exhibits an $E_g$ mode of bismuth which derived from the Bi bilayer in the middle of I-Bi-Bi-I four atomic layers in $Bi_xI_y$.

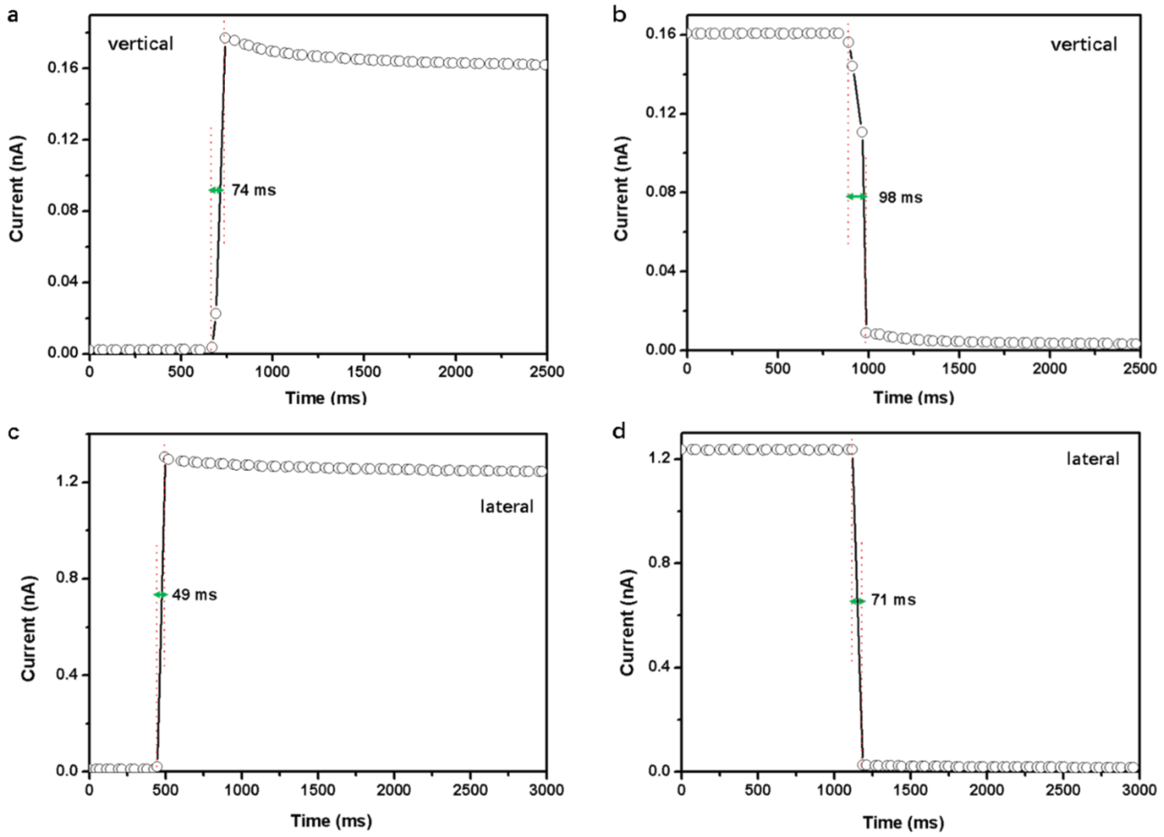


**Supplementary Figure 13 | X-ray response speed of $Bi_xI_y$. a-d,** Detailed on/off X-ray responses of $Bi_xI_y$ show rise/fall times of 49/71 ms for lateral device (**a**, **b**) and 74/98 ms for vertical device (**c**, **d**) under 1 V $mm^{-1}$ bias.

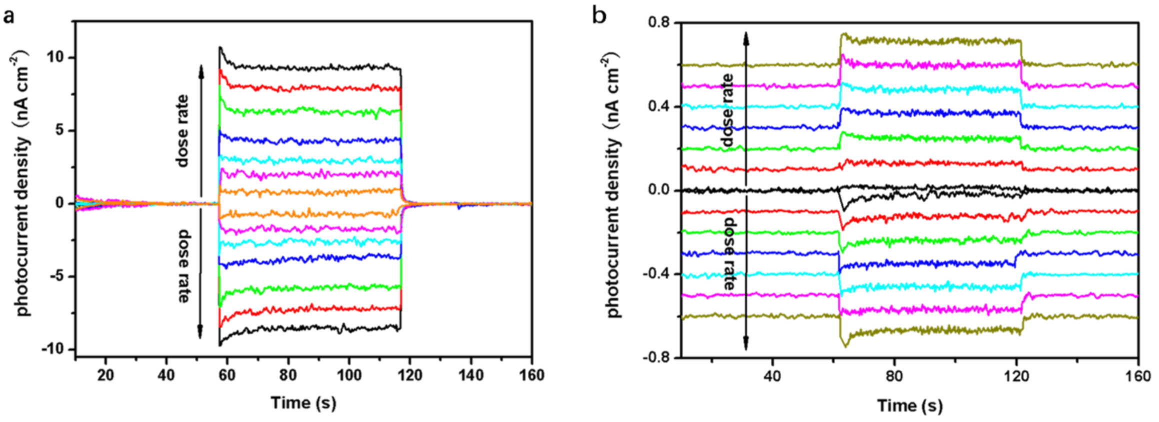


**Supplementary Figure 14 ｜Anisotropic X-ray responses of $Bi_xI_y$. a, b,** On/off X-ray responses of the lateral device **(a)** and vertical device **(b)** at different dose rates measured by cavity-edge configuration at room temperature.

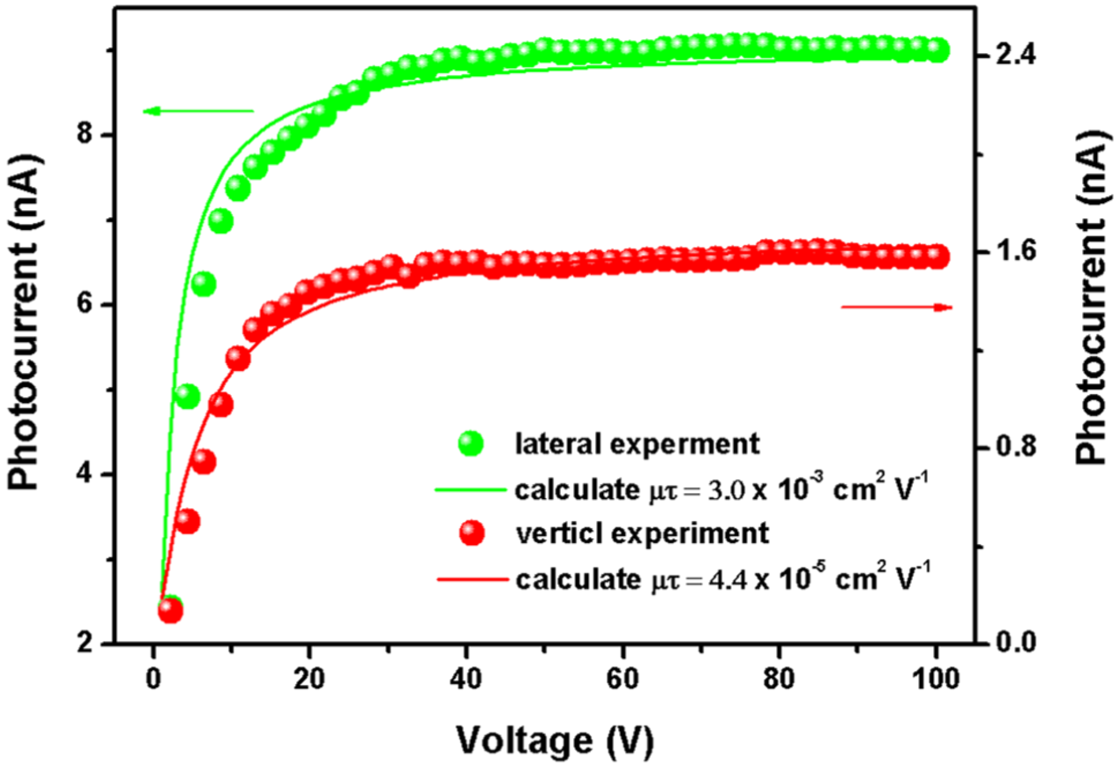


**Supplementary Figure 15 | Charge collection property of $Bi_xI_y$.** Anisotropic bias dependent photocurrent derived from the devices with $Si/Bi_xI_y/Ag$ configuration measured by intracavity configuration under dose rate with an X-ray tube current of 200 μA. The Si electrode was prepared by mixed the ground Si powders with the supernatant of silver paste. $\mu\tau$ product can be fitted by the Hecht equation:

$$I = \frac{I_0 \mu\tau V}{L^2}\left(1 - \exp\left(-\frac{L^2}{\mu\tau V}\right)\right)$$

Where $I_0$ is the saturated photocurrent, $L$ the thickness between electrodes (1 mm for lateral and 0.3 mm for vertical device) and $V$ the applied bias.

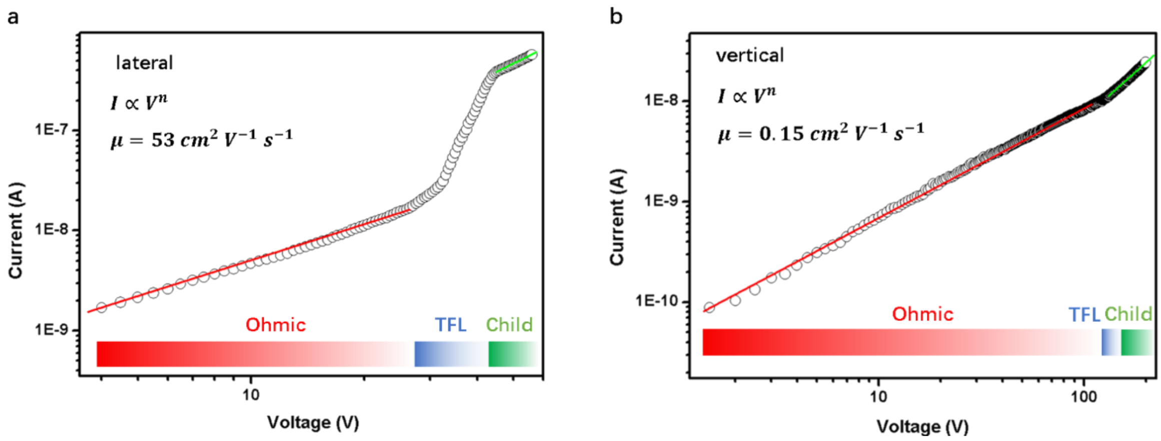


**Supplementary Figure 16 | Charge transport property of $Bi_xI_y$. a, b,** I–V curves of $Bi_xI_y$ at lateral **(a)** and vertical **(b)** directions with Ag/$Bi_xI_y$/Ag configuration fitted by the SCLC model. There were three distinct regions in the figure: an Ohmic region with n = 1, trap filling limited (TFL) region with n > 3, and a Child region with n = 2 appear in turn with increasing bias voltage. Charge carrier mobility μ is decided from the Child region by the Mott-Gurney law:

$$J = 9\varepsilon\varepsilon_0\mu V^2/8L^3$$

where $L$ is the distance between electrode (1 mm for lateral and 0.4 mm for vertical device), the relative dielectric constant of $BiI_3$ is obtained from supplementary ref. 2.

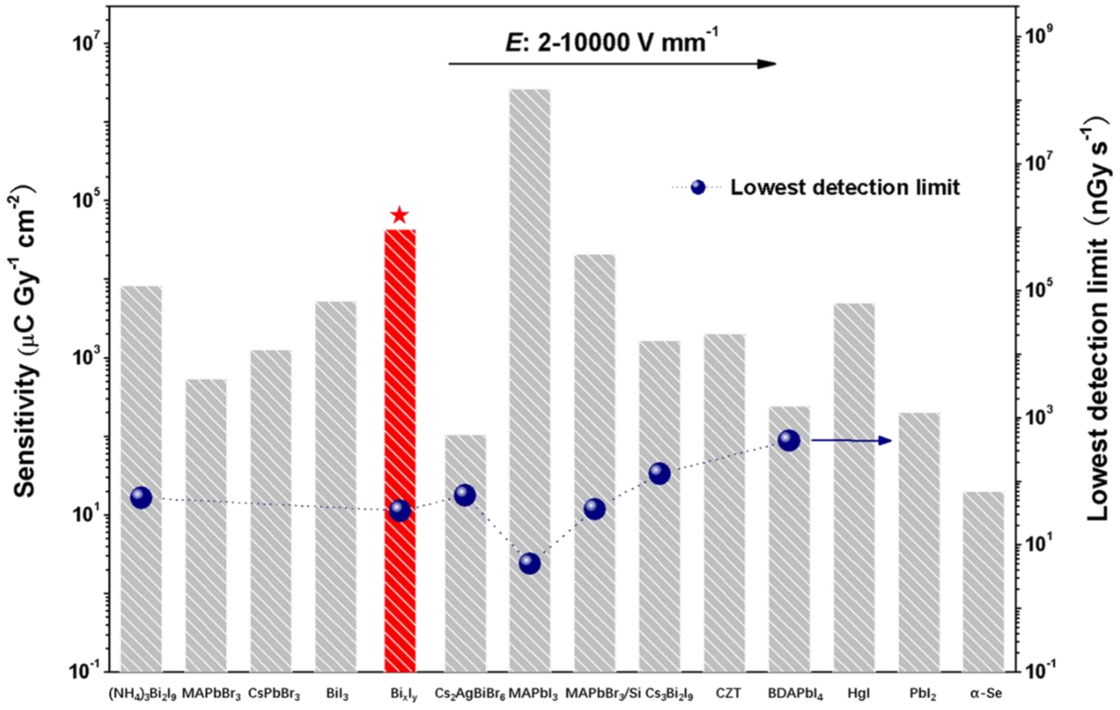


**Supplementary Figure 17 | Device comparison.** X-ray detection sensitivities and detection limits comparisons of the $Bi_xI_y$ detector in this work with some traditional (CdZnTe, $PbI_2$, $HgI_2$, $BiI_3$, α-Se) and recently reported ($MAPbBr_3$, $MAPbBr_3$/Si, $CsPbBr_3$, $Cs_2AgBiBr_6$, $MAPbI_3$, $BDAPbI_4$, $Cs_3Bi_2I_9$, $(NH_4)_3Bi_2I_9$) detectors. Data for CdZnTe, $PbI_2$, $HgI_2$ and $CsPbBr_3$ were extracted from supplementary ref. 3.

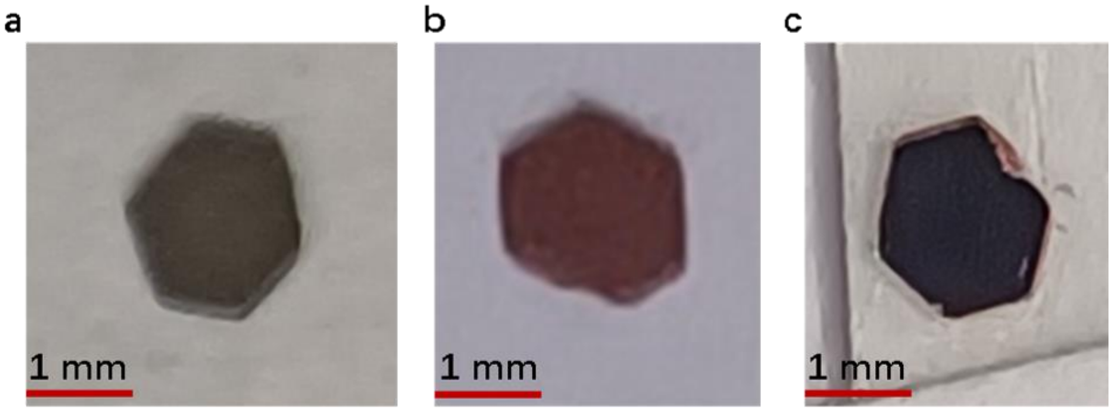


**Supplementary Figure 18 ｜Humidity stability of $Bi_xI_y$. a, b,** The images of $Bi_xI_y$ before (**a**) and after (**b**) 8 h water immersion. c, the interior image of $Bi_xI_y$ by separating the top layers after 8 h water immersion. The surface hydrolysis was observed after 8 h water immersion. The corrupted surface could be stripped and the interior remains unchanged.

## Supplementary references:


1 Hamdeh, U. H. *et al.* Solution-Processed $BiI_3$ Thin Films for Photovoltaic Applications: Improved Carrier Collection via Solvent Annealing. *Chem.Mater.* **28**, 6567-6574 (2016).

2 Du, M. H. *et al*. Enhanced Born charges in III-VII, IV-$VII_2$, and V-$VII_3$ compounds. *Phys. Rev. B* **82**, 045203 (2010).

3 Zhang, H. J. *et al*. High-sensitivity X-ray detectors based on solution-grown caesium lead bromide single crystals. *J. Mater. Chem. C* **8**, 1248-1256 (2020).
